# PLK1 (polo like kinase 1) inhibits MTOR complex 1 and promotes autophagy

**DOI:** 10.1080/15548627.2016.1263781

**Published:** 2017-01-19

**Authors:** Stefanie Ruf, Alexander Martin Heberle, Miriam Langelaar-Makkinje, Sara Gelino, Deepti Wilkinson, Carolin Gerbeth, Jennifer Jasmin Schwarz, Birgit Holzwarth, Bettina Warscheid, Chris Meisinger, Marcel A. T. M. van Vugt, Ralf Baumeister, Malene Hansen, Kathrin Thedieck

**Affiliations:** a Department of Bioinformatics and Molecular Genetics, Faculty of Biology, University of Freiburg, Freiburg, Germany; b Department of Pediatrics, Center for Liver, Digestive and Metabolic Diseases, University of Groningen, University Medical Center Groningen, AV Groningen, The Netherlands; c BIOSS Centre for Biological Signalling Studies, University of Freiburg, Freiburg, Germany; d Research Training Group (RTG) 1104, University of Freiburg, Freiburg, Germany; e Program of Development, Aging and Regeneration, Sanford Burnham Prebys Medical Discovery Institute, La Jolla, CA, USA; f Graduate School of Biomedical Sciences, Sanford Burnham Prebys Medical Discovery Institute, La Jolla, CA, USA; g ZBMZ Centre for Biochemistry and Molecular Cell Research (Faculty of Medicine), University of Freiburg, Freiburg, Germany; h Institute of Biochemistry and Molecular Biology (Faculty of Medicine), University of Freiburg, Freiburg, Germany; i Department of Biochemistry and Functional Proteomics, Faculty of Biology, University of Freiburg, Freiburg, Germany; j Spemann Graduate School of Biology and Medicine (SGBM), University of Freiburg, Freiburg, Germany; k Department of Medical Oncology, Cancer Research Center Groningen, University of Groningen, University Medical Center Groningen, GZ Groningen, The Netherlands; l Department for Neuroscience, School of Medicine and Health Sciences, Carl von Ossietzky University Oldenburg, Oldenburg, Germany

**Keywords:** amino acid, BI2536, insulin, interphase, lysosome, MTOR, MTORC1, PLK1, RPTOR, starvation

## Abstract

Mechanistic target of rapamycin complex 1 (MTORC1) and polo like kinase 1 (PLK1) are major drivers of cancer cell growth and proliferation, and inhibitors of both protein kinases are currently being investigated in clinical studies. To date, MTORC1′s and PLK1′s functions are mostly studied separately, and reports on their mutual crosstalk are scarce. Here, we identify PLK1 as a physical MTORC1 interactor in human cancer cells. PLK1 inhibition enhances MTORC1 activity under nutrient sufficiency and in starved cells, and PLK1 directly phosphorylates the MTORC1 component RPTOR/RAPTOR in vitro. PLK1 and MTORC1 reside together at lysosomes, the subcellular site where MTORC1 is active. Consistent with an inhibitory role of PLK1 toward MTORC1, PLK1 overexpression inhibits lysosomal association of the PLK1-MTORC1 complex, whereas PLK1 inhibition promotes lysosomal localization of MTOR. PLK1-MTORC1 binding is enhanced by amino acid starvation, a condition known to increase autophagy. MTORC1 inhibition is an important step in autophagy activation. Consistently, PLK1 inhibition mitigates autophagy in cancer cells both under nutrient starvation and sufficiency, and a role of PLK1 in autophagy is also observed in the invertebrate model organism *Caenorhabditis elegans*. In summary, PLK1 inhibits MTORC1 and thereby positively contributes to autophagy. Since autophagy is increasingly recognized to contribute to tumor cell survival and growth, we propose that cautious monitoring of MTORC1 and autophagy readouts in clinical trials with PLK1 inhibitors is needed to develop strategies for optimized (combinatorial) cancer therapies targeting MTORC1, PLK1, and autophagy.

## Introduction

PLK1 (polo like kinase 1) is a ubiquitously expressed serine/threonine protein kinase, which is widely recognized as an oncogene that drives cellular proliferation by promoting mitosis and cytokinesis.^1-3^ The 5 polo like kinase (PLK) family members PLK1 to 5 all contain a polo-box domain that regulates their kinase activity and subcellular localization.^1-3^ PLK1 is the best-described PLK protein, and is frequently used as a tumor marker, as high PLK1 expression correlates with poor prognosis in cancer.^4^ PLK1 inhibitors, such as BI2536, compete with adenosine triphosphate (ATP) for its binding to the catalytic domain of PLK1.^5^ Long-term PLK1 inhibition arrests cells in prometaphase, and thus PLK1 inhibitors are investigated as antimitotic agents for cancer treatment.^1,6,7^ MTOR (mechanistic target of rapamycin) is another serine/threonine protein kinase that promotes cellular growth and is also often targeted in cancer therapy.^8,9^ Although both PLK1^2,3^ and MTOR^10^ are conserved in invertebrates and mammals, little is known about their crosstalk and mutual regulation of common downstream processes, as well as the implications thereof for cancer therapies.

The nutrient sensor MTOR is activated by metabolic stimuli, including amino acids, growth factors (e.g., insulin), and energy sufficiency.^10-12^ MTOR acts in 2 structurally and functionally distinct multiprotein complexes, MTOR complex 1 (MTORC1) and MTORC2.^10,12^ RPTOR/RAPTOR (regulatory associated protein of MTOR complex 1) is a core component of MTORC1,^10,12^ which is a central controller of cellular growth and survival. Consistently, MTORC1 is dysregulated in many cancer types,^8^ and several compounds for pharmacological MTORC1 inhibition are investigated as cancer therapeutics.^8,9^ The MTORC1-specific allosteric inhibitor rapamycin and its analogs (rapalogs) are already approved for the treatment of several tumor entities.^9^ The more recently developed ATP-analog MTOR inhibitors, such as Torin1^13^ and its derivatives, are currently tested in clinical studies.^9^ They target both MTOR complexes, and also inhibit MTORC1 functions which are insensitive to rapamycin.^13^ Amino acid- and growth factor- induced signaling pathways converge at the lysosomes to synergistically activate MTORC1.^11^ MTORC1 activation by amino acids requires RAG GTPase-mediated MTORC1 translocation to lysosomes.^11,14,15^ Conversely, loss of lysosomal MTORC1 association mediates MTORC1 inhibition upon amino acid withdrawal.^16^ At the lysosome, MTORC1 encounters the small GTPase Ras homolog enriched in brain (RHEB),^11,17^ which activates MTORC1 downstream of the INSR (insulin receptor)-phosphoinositide 3-kinase-AKT signaling axis.^10,11,18^ RHEB is inhibited by the heteromeric TSC1-TSC2 (tuberous sclerosis 1 and 2) complex, which acts as a GTPase-activating protein (GAP) on RHEB. MTORC1 phosphorylates several substrates^19^ that mediate its anabolic outcomes. Among them is RPS6KB (p70)/p70-S6K (ribosomal protein S6 kinase B 70 kDa) which is phosphorylated at threonine 389 (T389) by MTORC1.^10,18,20^ In turn, RPS6KB (p70) activates protein synthesis by promoting expression of ribosomal components,^21^ and by phosphorylating translation initiation factors and components of the ribosomal machinery, including RPS6 (ribosomal protein S6).^19^ Little is known about PLK1′s role in the MTORC1 pathway. Even though several studies correlate PLK1 inhibition with either decreased^22-25^ or increased^26^ RPS6KB (p70) or RPS6 phosphorylation, a clear functional interaction between PLK1 and MTORC1 has so far not been reported. Thus, it is unknown whether PLK1 regulates phosphorylation of MTORC1 substrates indirectly or directly, i.e., by physically acting on MTORC1.

MTORC1 promotes cellular growth by inducing anabolic processes including protein synthesis, and by inhibiting catabolic processes.^10,19^ Conversely, MTORC1 inhibition derepresses catabolic processes to promote cellular survival, e.g., when nutrients are scarce.^10^ The best-described catabolic process inhibited by MTORC1 is autophagy, and this MTORC1 function is conserved from yeast and invertebrates such as *Caenorhabditis elegans**^27^* (*C. elegans*) to mammals.^28^ Autophagy is tightly balanced to maintain cellular homeostasis and fuel cells with nutrients and metabolite intermediates under nutrient sufficiency and deprivation^29^ via degradation of proteins, lipids, and organelles in the lysosomes.^28,30^ Macroautophagy (from here on referred to as autophagy) is to date the best-characterized type of autophagy.^30^ During autophagy, double-membrane vesicles called autophagosomes are formed which fuse with late endosomes or lysosomes to form autolysosomes, in which the degradation of the sequestered material takes place.^30,31^ In the context of cancer, autophagy gains growing attention as autophagy contributes to the elimination of tumor cells, but also promotes tumor survival.^32-34^ Consequently, both autophagy inhibitors, such as chloroquine,^35^ and autophagy activators, e.g., proteasome and MTORC1 inhibitors,^33,34^ are currently investigated in clinical trials. Of note, ATP-analog MTOR inhibitors such as Torin1 enhance autophagy more effectively than rapalogs, as ATP analogs block autophagy-inhibiting MTORC1 functions that are rapamycin resistant.^13^ Autophagy is also regulated by multiple MTORC1-independent cues.^28^ For example, during mitosis autophagy is inhibited in an MTORC1-independent manner.^36,37^ Links of PLK1 with autophagy are poorly explored. PLK1 is known to localize to centrosomes, kinetochores, and the mitotic spindle,^2^ and PLK1 expression is increased during mitosis.^38^ During this cell cycle phase PLK1 has been suggested to contribute to autophagy inhibition.^39,40^ As PLK1 research mostly focuses on mitotic cells, it is unknown whether PLK1 affects autophagy in interphase cells and which signaling networks might mediate such effects. Such knowledge would broaden the range of application of PLK1 inhibitors specifically to tumors that display low mitotic rates,^41^ and/or require autophagy for cellular growth and survival.^42^ It would also reveal potential effects of PLK1 inhibitors on MTOR and autophagy networks that may be relevant for therapy outcome. Therefore, we analyzed in the present study whether and what type of crosstalk exists between PLK1, MTORC1, and autophagy in nonmitotic cancer cells.

We describe here a novel nonmitotic function of PLK1. We identify PLK1 as a physical interactor of MTORC1, whose scaffold component RPTOR is a direct PLK1 substrate in vitro. We find that PLK1 inhibition leads to hyperphosphorylation of the MTORC1 substrate RPS6KB (p70). PLK1 resides with MTORC1 at lysosomes, a localization hitherto unknown for PLK1; and the PLK1-MTORC1 complex colocalizes with and physically binds LAMP2 (lysosomal-associated membrane protein 2). Consistent with an inhibitory function of PLK1 toward MTORC1, overexpression of active PLK1 detaches the PLK1-MTORC1 complex from the lysosomes, and PLK1 inhibition increases MTOR localization at lysosomes. In keeping with this, PLK1 inhibition mitigates autophagy in both the invertebrate model organism *C. elegans*, and in mammalian cells, where autophagy is regulated in an MTORC1-dependent manner. In conclusion, PLK1 positively contributes to autophagy via inhibition of MTORC1 under nutrient sufficiency and starvation. Our findings highlight the importance of carefully monitoring PLK1-, MTOR-, and autophagy- activities in clinical studies, to identify leads for cancer therapy design.

## Results

### PLK1 physically interacts with MTOR and RPTOR

**Figure 1.**
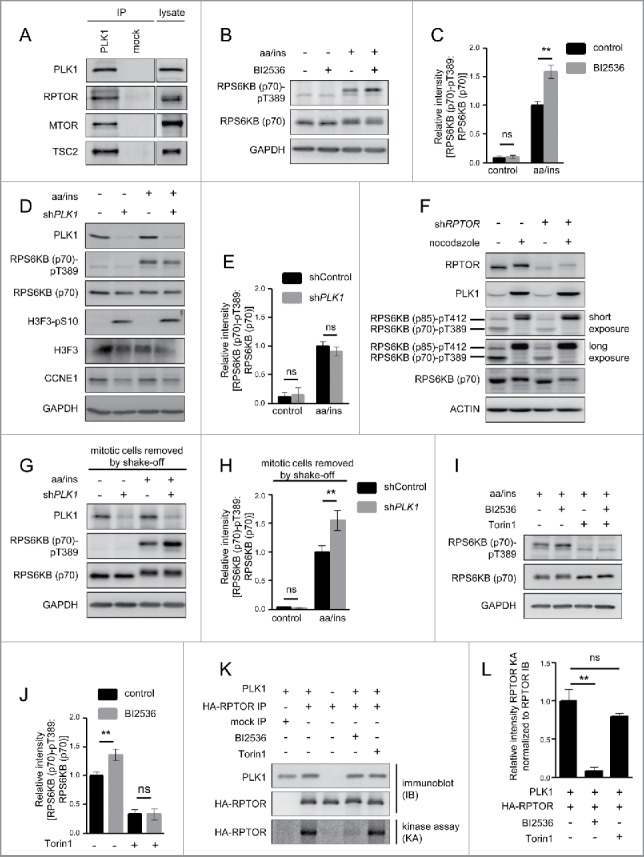
PLK1 binds and phosphorylates MTORC1, and PLK1 inhibition activates MTORC1 in interphase cells. (A) HeLa cells were cultured in full medium. Immunoprecipitation (IP) was performed with PLK1 and control (mock) antibodies. Samples were analyzed by immunoblotting. Data shown are representative of n = 4 independent experiments. (B) HeLa cells were starved for 1 h for amino acids and growth factors, stimulated with amino acids and insulin for 35 min and treated with the PLK1 inhibitor BI2536 for 30 min, as indicated. Samples were analyzed by immunoblotting. Data shown are representative of n = 3 independent experiments. (C) Quantification of data shown in (B). Ratio of RPS6KB (p70) phospho-(T389)/RPS6KB (p70) was calculated for n = 3 independent experiments. Data are normalized to 1 for the amino acid- and insulin-stimulated control condition, and represented as mean ± SEM. A one-way ANOVA followed by the Bonferroni multiple comparison test was applied; ns, nonsignificant; **, *P* ≤ 0.01. (D) *PLK1* (sh*PLK1*) or control (shControl) shRNA HeLa cells were starved for amino acids and growth factors for 1 h, and stimulated with amino acids and insulin for 30 min. Samples were analyzed by immunoblotting without removal of the mitotic cells. Data shown are representative of n = 3 independent experiments. (E) Quantification of data shown in (D). Ratio of RPS6KB (p70) phospho-(T389):RPS6KB (p70) was calculated for n = 3 independent experiments. Data are normalized to 1 for the amino acid- and insulin-stimulated shControl condition, and represented as mean ± SEM. A one-way ANOVA followed by the Bonferroni multiple comparison test was applied; ns, nonsignificant. (F) *RPTOR* shRNA (sh*RPTOR*) or shControl HeLa cells were arrested in mitosis by nocodazole treatment. Samples were analyzed by immunoblotting. Data shown are representative of n = 3 independent experiments. (G) *PLK1* (sh*PLK1*) or control (shControl) shRNA HeLa cells were starved for amino acids and growth factors for 16 h and stimulated with amino acids and insulin for 35 min. Mitotic cells were removed by shake-off. Samples were analyzed by immunoblotting. Data are representative of n = 4 independent experiments. (H) Quantification of data shown in (G). Ratio of RPS6KB (p70) phospho-(T389):RPS6KB (p70) was calculated for n = 4 independent experiments. Data are normalized to 1 for the amino acid- and insulin-stimulated shControl condition and represented as mean ± SEM. A one-way ANOVA followed by the Bonferroni multiple comparison test was applied; ns, nonsignificant; **, *P* ≤ 0.01. (I) HeLa cells were treated with BI2536 and/or Torin1 as indicated, and stimulated as described in (B). Samples were analyzed by immunoblotting. Data shown are representative of n = 3 independent experiments. (J) Quantification of data shown in (I). Ratio of RPS6KB (p70) phospho-(T389):RPS6KB (p70) was calculated for n = 3 independent experiments. Data are normalized to 1 for control condition (no Torin1, no BI2536), and represented as mean ± SEM. A one-way ANOVA followed by the Bonferroni multiple comparison test was applied; ns, nonsignificant; **, *P* ≤ 0.01. (K) PLK1 kinase assay. HA-RPTOR was immunopurified from HeLa cells. An unspecific IgG antibody was used as negative control. All samples were dephosphorylated before adding them to the kinase reaction with recombinant PLK1. Data shown are representative of n = 3 independent experiments. IP, immunoprecipitation; IB, immunoblot; KA, kinase assay. (L) Quantification of data shown in (K) for n = 3 independent experiments. Data are normalized to 1 for HA-RPTOR phosphorylation by PLK1. Data are represented as mean ± SEM. A one-way ANOVA followed by the Bonferroni multiple comparison test was applied; ns, nonsignificant; **, *P* ≤ 0.01. (B, C, D, E, G, H, I) aa, amino acids; ins, insulin.

We have recently analyzed the MTOR interactome by quantitative proteomics.^43^ In this study,^43^ we purified endogenous MTOR kinase by immunoprecipitation (IP) from the cervical cancer cell line HeLa, and analyzed MTOR IPs versus mock IPs, conducted with a nonspecific control IgG. We reanalyzed those data here, and found that PLK1 was specifically identified by tandem mass spectrometry in MTOR IPs for 2 out of 3 biological replicates (Schwarz et al, Table S4^43^) with 6 peptides and a sequence coverage of 11% (Fig. S1A). Annotated MS1 and fragment spectra for one of the PLK1 peptides are shown in Fig. S1B and S1C. Physical interaction of PLK1 with MTOR has not been reported previously. To validate this finding, we performed PLK1 and mock IPs and analyzed them by immunoblotting (Fig. 1A, S1D). TSC2 and the MTORC2 component RICTOR (RPTOR-independent companion of MTOR complex 2) were specifically detected in PLK1 IPs, serving as positive controls, as interaction of TSC2 and RICTOR with PLK1 has been shown earlier.^23,44^ Of note, we also specifically detected MTOR and the MTORC1 component RPTOR in the PLK1 IP, but not in the mock IP (Fig. 1A). To test if RPTOR is required for PLK1-MTORC1 binding, we immunoprecipitated PLK1 from lysates of stably transduced HeLa cells with doxycycline-inducible expression constructs for short hairpin RNAs targeting *RPTOR* (sh*RPTOR*),^45^ or harboring a nontargeting sequence (shControl). PLK1 bound MTOR to the same extent in sh*RPTOR* or shControl knockdown cells (Fig. S1E, S1F), suggesting that PLK1 physically binds MTORC1 via MTOR.

### PLK1 inhibits MTORC1 in nonmitotic cells

Next, we investigated whether PLK1 influences MTORC1 activity. We tested this first upon MTORC1 activation with amino acids and insulin. To inhibit PLK1, we treated HeLa cells for 30 min with the ATP-competitive PLK1 inhibitor BI2536.^5^ We combined the PLK1 inhibitor treatment with amino acid and insulin stimulation, and analyzed phosphorylation of RPS6KB (p70) at T389 as a bona fide readout for MTORC1 activity. As expected, immunoblotting showed that amino acid and insulin stimulation increased RPS6KB (p70) T389 phosphorylation, consistent with MTORC1 activation (Fig. 1B, first vs third lane). Treatment with the PLK1 inhibitor BI2536 further enhanced RPS6KB (p70) T389 phosphorylation significantly (Fig. 1B, third vs fourth lane; 1C). Thus, PLK1 inhibition leads to RPS6KB (p70) hyperphosphorylation at T389 upon stimulation with amino acids and insulin, suggesting that PLK1 inhibits MTORC1.

To confirm this result by another mode of PLK1 inhibition and to control for possible off-target effects of the PLK1 inhibitor BI2536, we next inhibited *PLK1* by RNA interference (RNAi). To this end, we stably transduced HeLa cells with doxycycline-inducible expression constructs for shRNAs targeting *PLK1* (sh*PLK1*), or a nontargeting sequence (shControl). Knockdown was induced by doxycycline treatment for 2 d. Surprisingly, we observed no change in RPS6KB (p70) T389 phosphorylation in sh*PLK1* as compared with shControl cells (Fig. 1D, E). This seemed contradictory to the increase in RPS6KB (p70) phosphorylation at T389 that we observed upon BI2536 treatment (Fig. 1B, C).

A main difference between BI2536- versus sh*PLK1*-treated cells was that the treatment with the inhibitor was done for a brief interval (i.e., 30 min), whereas sh*PLK1* treatment was performed for 2 d, which was required to achieve efficient PLK1 knockdown. During these 2 d, we observed an increasing amount of rounded and detached cells, probably due to elevated numbers of mitotic cells, as long-term PLK1 inhibition leads to mitotic arrest.^46,47^ We thus hypothesized that the difference in RPS6KB (p70) T389 phosphorylation in sh*PLK1*- versus BI2536-treated cells could result from a larger fraction of mitotic cells in sh*PLK1* cultures, or from differing (off-target) effects during sh*PLK1* or BI2536 treatment. To test the first possibility directly, we analyzed if mitotic markers were increased in sh*PLK1*- and/or BI2536-treated cells. In sh*PLK1*-treated cells, we observed increased phosphorylation of the mitotic marker H3F3/histone H3 (H3 histone family member 3) at serine 10 (S10), and decreased levels of the G_1_/S phase marker CCNE1 (cyclin E1), indicative of an increased mitotic cell fraction in sh*PLK1* cultures (Fig. 1D). In contrast, short-term treatment with the PLK1 inhibitor BI2536 did not lead to an apparent increase in H3F3 S10 phosphorylation (Fig. S2A). As a positive control, the H3F3 phospho-(S10) antibody was in parallel used to detect a cell lysate of mitotic cells (Fig. S2A), and showed a strong signal. In agreement with earlier studies,^3,46,47^ long-term overnight BI2536 treatment enhanced H3F3 phosphorylation at S10 (Fig. S2B). Thus, we conclude that short-term BI2536 treatment failed to cause a detectable shift in cell cycle distribution, whereas long-term sh*PLK1* induction did. This may be the reason for the observed differences in MTORC1 signaling between these 2 experimental setups.

To further test this, we aimed to separate effects directly mediated by PLK1 from its indirect, mitotic arrest-related effects. For this purpose, we first analyzed RPS6KB (p70) phosphorylation in mitotic versus asynchronous cell cultures, with or without MTORC1 inhibition by sh*RPTOR* (Fig. 1F). We arrested cells in prometaphase by nocodazole treatment, followed by a mitotic shake-off to enrich for mitotic cells. Immunoblot analysis showed that PLK1 levels were increased in nocodazole plus shake-off-treated cells, indicative of a mitotic arrest.^38^ Phosphorylation of the p70 isoform RPS6KB (p70) at T389 was observed in asynchronous cells, but not in cells with mitotic arrest, indicating that MTORC1 is inactive in mitotic cells (Fig. 1F). Interestingly, phosphorylation of the p85 isoform RPS6KB (p85) at T412^48^ [RPS6KB (p85) phospho-(T412), which is detected by the same antibody as RPS6KB (p70) phospho-(T389) and thus appears at a higher molecular weight in the same blot] was enhanced in mitotically arrested cells compared with nonarrested cells (Fig. 1F, first vs. second lane). This induction of phospho-RPS6KB (p85) at T412 possibly explains earlier reports on MTORC1 activation in mitosis.^49^ In contrast, T412 phosphorylation of RPS6KB (p85) in nocodazole-arrested cells was not inhibited by shRNA-mediated knockdown of the MTORC1 component RPTOR (Fig. 1F, fourth vs second lane), indicating that a kinase other than MTOR as member of MTORC1 mediates this event. In contrast, sh*RPTOR* did reduce the signal for phospho-RPS6KB (p70) at T389 in asynchronous cells (Fig. 1F, first vs third lane). Thus, the absence of phospho-RPS6KB (p70) signal at T389 in prometaphase-arrested cells suggests that MTORC1 is inhibited in mitosis (Fig. 1F, second and fourth lane), which is in line with previous findings.^50^ This supports our hypothesis that an increase in the amount of mitotic cells in a culture, as observed after PLK1 knockdown, may mask MTORC1 activation in the nonmitotic cell fraction in the same culture.

To test this, we combined PLK1 knockdown with removal of mitotic cells by shake-off. The removal of the mitotic cells was efficient, as evidenced by the decline in H3F3 S10 phosphorylation in cultures after shake-off, compared with those without shake-off (Fig. S2C, fourth vs. third lane). In the nonmitotic cells that remained in the culture after shake-off, PLK1 knockdown did significantly increase RPS6KB (p70) phosphorylation at T389 in response to amino acid and insulin stimulation (Fig. 1G, H) to a similar extent as BI2536 (Fig. 1B, C). Thus, both BI2536 and sh*PLK1* treatments in nonmitotic cells yielded qualitatively and quantitatively similar results, namely RPS6KB (p70) phospho-(T389) induction. This suggests that PLK1 acts to inhibit MTORC1 in nonmitotic cells. To test whether enhanced RPS6KB (p70) phospho-(T389) in PLK1-inhibited cells is consistent with MTORC1 activation, we combined PLK1 inhibition by BI2536 with MTOR inhibition by Torin1.^13^ Torin1 reduced RPS6KB (p70) T389 phosphorylation both in control and BI2536-treated cells (Fig. 1I, J), consistent with the notion that increased RPS6KB (p70) phosphorylation at T389 in PLK1-inhibited cells is mediated by MTOR.

Taken together, RPS6KB (p70) was hyperphosphorylated at T389 when PLK1 was blocked pharmacologically or through shRNA in nonmitotic cells. This suggests that PLK1 inhibits MTORC1 and limits the extent of RPS6KB (p70) T389 phosphorylation in response to nutrients and insulin in interphase cells.

### PLK1 phosphorylates the MTORC1 component RPTOR in vitro

We found that PLK1 physically interacts with MTOR and its specific binding partner RPTOR (Fig. 1A), and that PLK1 inhibition activates MTORC1 in amino acid- and insulin-stimulated cells (Fig. 1B, G). Therefore, we next tested whether MTORC1 can function as a direct PLK1 substrate in vitro. The MTORC1 component RPTOR acts as a scaffold for the binding of MTORC1's substrates^10^ and is required for MTORC1 activity.^51,52^ RPTOR is targeted by several kinases that signal to MTORC1,^10^ for example, AMPK (AMP-activated protein kinase)^10^ and RPS6KA1/RSK (ribosomal protein S6 kinase A1).^53^ To test whether PLK1 is also capable of phosphorylating RPTOR, we overexpressed and immunopurified HA-tagged RPTOR from HeLa cells and used it as a substrate for in vitro kinase assays with recombinant PLK1 and ^33^P-labeled ATP (Fig. 1K). We detected incorporation of ^33^P at the molecular weight of HA-RPTOR, and this signal was reduced by the PLK1 inhibitor BI2536 (Fig. 1K, L). Thus, the observed HA-RPTOR phosphorylation was PLK1-specific. The MTOR inhibitor Torin1 did not significantly reduce the radioactive HA-RPTOR signal (Fig. 1K, L), suggesting that MTOR background activity does not contribute to the signal. As a negative control we omitted either PLK1 or HA-RPTOR from the in vitro kinase reaction. In both cases, no radioactive signal was detected at the molecular weight of HA-RPTOR (Fig. 1K, first and third lane), showing that the signal is RPTOR specific and requires the presence of PLK1. Thus, we conclude that PLK1 can directly phosphorylate RPTOR *in vitro*.

### PLK1 resides with MTORC1 at lysosomes, and active PLK1 decreases lysosomal association of the PLK1-MTORC1 complex

**Figure 2.**
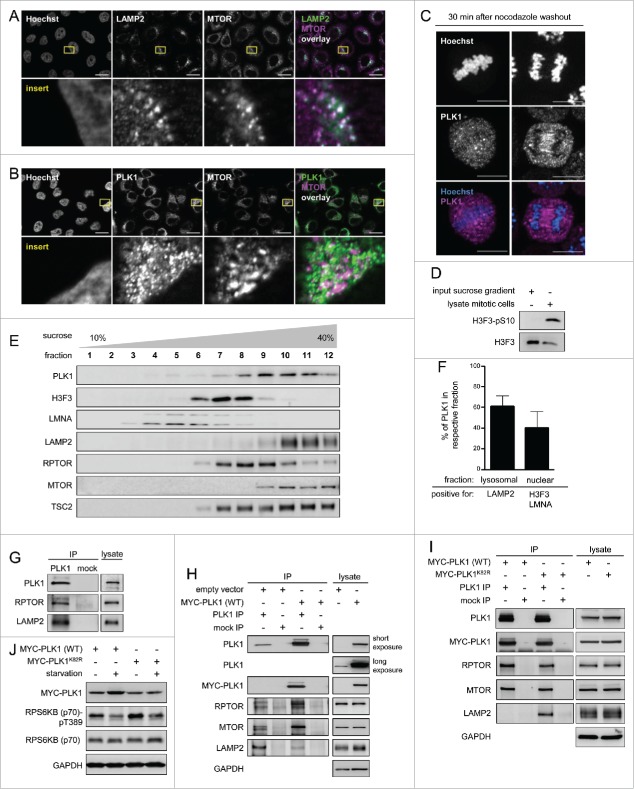
PLK1 resides with MTORC1 at lysosomes, and overexpression of active PLK1 decreases lysosomal association of the PLK1-MTORC1 complex. (A) Immunofluorescence analysis of HeLa cells that were cultured in full medium and stained with LAMP2 and MTOR antibodies. White regions in merged image (right) of LAMP2 (green) and MTOR (magenta) indicate colocalization. Nuclei were stained with Hoechst 33342. Scale bar 20 µm. Representative images are shown for n = 3 independent experiments. (B) Immunofluorescence analysis of HeLa cells that were cultured in full medium and stained with PLK1 and MTOR antibodies. White regions in merged image (right) of PLK1 (green) and MTOR (magenta) indicate colocalization. Nuclei were stained with Hoechst 33342. Scale bar 20 µm. Representative images are shown for n = 3 independent experiments. (C) Immunofluorescence analysis of HeLa cells that were synchronized in prometaphase with nocodazole for 16 h and released for 30 min in full medium. Cells were stained with PLK1 antibody. Nuclei were stained with Hoechst 33342. Scale bar: 10 µm. Representative images of cells in metaphase (left) and anaphase (right) are shown for n = 3 independent experiments. (D) Analysis of input sample taken before fractionation in sucrose gradient (E). The mitotic cell lysate was collected from HeLa sh*PLK1* knockdown cultures without mitotic shake-off. Samples were analyzed by immunoblotting. Data shown are representative of n = 2 independent experiments. (E) HeLa cells were starved for 1 h for amino acids and growth factors and stimulated with amino acids and insulin for 35 min. Samples were separated in a 10 to 40% sucrose gradient and analyzed by immunoblotting. Data shown are representative of n = 3 independent experiments. (F) Quantification of data shown in (E) for n = 3 independent experiments. The percentage of PLK1 in either the lysosomal or the nuclear fraction is displayed. Data are represented as mean ± SEM. (G) HeLa cells were cultured in full medium. Immunoprecipitation (IP) was performed with PLK1 and control (mock) antibodies. Samples were analyzed by immunoblotting. Data shown are representative of n = 3 independent experiments. (H) HeLa cells overexpressing wild type MYC-PLK1 (WT) or empty vector were cultured in full medium. Immunoprecipitation (IP) was performed with PLK1 and control (mock) antibodies. Samples were analyzed by immunoblotting. Data shown are representative of n = 3 independent experiments. (I) HeLa cells overexpressing MYC-PLK1 (WT) or kinase-defective, dominant negative MYC-PLK1^K82R^ were cultured in full medium. Immunoprecipitation (IP) was performed with PLK1 and control (mock) antibodies. Samples were analyzed by immunoblotting. Data shown are representative of n = 3 independent experiments. (J) HeLa cells overexpressing MYC-PLK1 (WT) or kinase-defective, dominant negative MYC-PLK1^K82R^ were starved for 1 h for amino acids and growth factors, and stimulated with amino acids and insulin for 35 min. Cells were then starved for amino acids for 10 min as indicated, and samples were analyzed by immunoblotting. Data shown are representative of n = 3 independent experiments.

Since PLK1 binds and can directly phosphorylate MTORC1, at least in vitro, we next asked in which common subcellular compartment they reside. In line with its function as a mitotic regulator, PLK1 localizes to multiple mitosis-specific structures, including centrosomes, kinetochores, and the spindle midzone,^2,3^ but also to the Golgi.^54^ Lysosomal localization is well described to be required for MTORC1 activation by amino acids and insulin,^14,15^ although MTOR localizes also to various other compartments.^17,55,56^ Localization of PLK1 to the lysosome has to the best of our knowledge so far not been reported. To test whether in nonmitotic cells PLK1 resides with MTORC1 at lysosomes, we first analyzed the localization of PLK1, MTOR and the lysosomal marker LAMP2 by immunofluorescence (IF) in unsynchronized HeLa cells (Fig. 2A, B). Consistent with MTOR's known localization at lysosomes,^14^ there was a strong overlap of MTOR and LAMP2 staining (Fig. 2A). In addition, PLK1 and MTOR colocalized with each other in a lysosomal pattern (Fig. 2B), suggesting that they reside together at a common subcellular site. We tested the specificity of the PLK1 antibody in mitotic metaphase and anaphase cells, where it detected PLK1 at the mitotic spindle, as reported^57^ (Fig. 2C). It was not experimentally possible to perform PLK1 and LAMP2 costaining as the antibodies against both PLK1 and LAMP2 were raised in mice and antibodies suitable for IF from other species were not available. To further test whether PLK1 localizes to lysosomes, we used sucrose gradients to fractionate cell lysates from unsynchronized HeLa cell cultures. The mitotic marker H3F3 phospho-(S10) was undetectable in these cultures, as compared with lysates from mitotically arrested HeLa cells (Fig. 2D), suggesting that mitotic cells in the unsynchronized cultures were below the detection threshold. As expected, distribution of endogenous PLK1 partially overlapped with fractions that contained the nuclear markers LMNA (lamin A/C) and H3F3. However, much stronger signals for PLK1 were found in fractions that were positive for the lysosomal marker LAMP2, MTOR, and RPTOR (Fig. 2E, F). Thus, PLK1 coresided with MTOR and RPTOR in the lysosomal fractions, suggesting that PLK1 may bind to lysosomes. To test whether PLK1 indeed physically interacts with lysosomal components, we analyzed PLK1 IPs with a LAMP2 antibody. Indeed, the lysosomal marker LAMP2 and the MTORC1 component RPTOR (positive control, see also Fig. 1A) were specifically detected in PLK1 IPs, but not in mock IPs (Fig. 2G), suggesting that PLK1 resides together with MTORC1 at lysosomes.

As our previous data (Fig. 1B, C, G to J) suggested that PLK1 inhibits MTORC1, and lysosomal relocalization is an important mode of MTORC1 regulation,^14-16,58^ we next tested whether PLK1 induction alters LAMP2-association of the PLK1-MTORC1 complex. To this end, we transfected cells with MYC-tagged, wild-type PLK1 [MYC-PLK1 (WT)]^38^ or an empty-control vector, performed PLK1 IPs, and detected LAMP2, MTOR and RPTOR by immunoblotting (Fig. 2H). MYC-PLK1 (WT) overexpression did not alter the endogenous MTOR, RPTOR, or LAMP2 levels in the lysates. Of note, MYC-PLK1 (WT) overexpression strongly enhanced MTOR and RPTOR signals in PLK1 IPs, whereas the LAMP2 signal was strongly decreased (Fig. 2H). As endogenous MTOR and RPTOR levels were unaltered in the lysates (Fig. 2H), our data suggest that there is an increase in the amount of PLK1-MTORC1 complexes upon MYC-PLK1 (WT) expression, and these complexes do not physically bind the lysosomal marker LAMP2. Thus, our data are consistent with a model in which active PLK1 dissociates MTORC1 from lysosomes. To test whether its kinase activity is required for overexpressed PLK1 to detach MTORC1 from LAMP2, we transfected cells either with MYC-PLK1 (WT), or with a dominant-negative lysine 82 to arginine mutated PLK1 variant (MYC-PLK1^K82R^),^59^ and performed PLK1 IPs (Fig. 2I). Endogenous MTOR, RPTOR, and LAMP2 levels were similar in lysates from MYC-PLK1 (WT) or MYC-PLK1^K82R^ transfected cells. In PLK1 IPs, the amounts of coimmunoprecipitated MTOR and RPTOR were similar for overexpression of MYC-PLK1 (WT) or dominant-negative MYC-PLK1^K82R^. In contrast, LAMP2 signals were stronger in PLK1 IPs from cells overexpressing dominant negative MYC-PLK1^K82R^, as compared with MYC-PLK1 (WT)(Fig. 2I). This suggests that inactive MYC-PLK1^K82R^ binds MTORC1 and the lysosomal protein LAMP2. Active MYC-PLK1 (WT) loses LAMP2 association while it binds MTORC1 with the same efficiency as inactive MYC-PLK1^K82R^ (Fig. 2I). In summary, these data are consistent with the notion that PLK1 binds MTORC1 at lysosomes, and that active PLK1 dissociates the PLK1-MTORC1 complex from the lysosomes, thereby mediating MTORC1 inhibition. If this was the case, a decrease in MTORC1 activity would be expected following overexpression of wild type PLK1 as compared with inactive PLK1. To test this, we analyzed RPS6KB (p70) T389 phosphorylation in starved or amino acid- and insulin-stimulated cells that were transfected with MYC-PLK1 (WT) or inactive MYC-PLK1^K82R^ (Fig. 2J). Consistent with an inhibitory function of active PLK1 toward MTORC1, RPS6KB (p70) phospho-(T389) induction by amino acids plus insulin was lower in MYC-PLK1 (WT)-transfected cells compared with MYC-PLK1^K82R^ transfected cells. Thus, we conclude that active MYC-PLK1 (WT) reduces lysosomal association of the PLK1-MTORC1 complex, which correlates with decreased RPS6KB (p70) T389 phosphorylation. This indicates that decreased lysosomal association contributes to MTORC1 inhibition by PLK1.

### PLK1 inhibition reduces autophagy in an MTORC1-dependent manner in interphase cells

**Figure 3.**
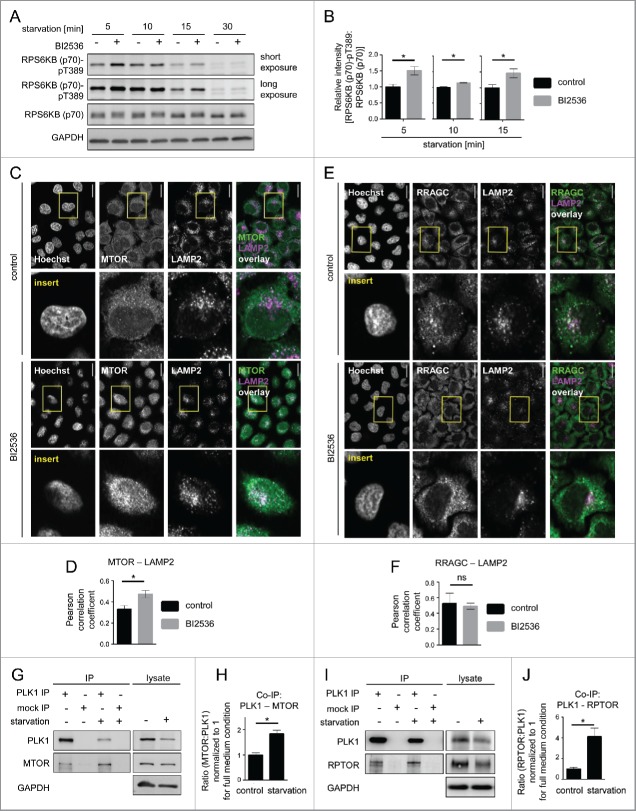
PLK1 inhibition hyperactivates MTORC1 and increases lysosomal MTORC1 localization in amino acid-starved interphase cells. (A) HeLa cells were starved for 1 h for amino acids and growth factors, and stimulated with amino acids and insulin for 35 min. Cells were then starved for amino acids for 5, 10, 15 and 30 min and treated with BI2536 or carrier, as indicated. Samples were analyzed by immunoblotting. Data shown are representative of n = 4 independent experiments. (B) Quantification of data shown in (A). Ratio of RPS6KB (p70) phospho-(T389):RPS6KB (p70) was calculated for n = 4 (5 min starvation and 15 min starvation); n = 3 (10 min starvation) independent experiments. Data are normalized to 1 for starvation control condition and represented as mean ± SEM. A nonparametric 2-tailed Student *t* test was applied; *, *P* ≤ 0.05. (C) Immunofluorescence analysis of HeLa cells that were starved for 1 h for amino acids and growth factors, stimulated with amino acids and insulin for 35 min, followed by 30 min amino acid starvation, without or with the PLK1 inhibitor BI2536. Staining was performed with MTOR and LAMP2 antibodies. White regions in merged image (right) of MTOR (green) and LAMP2 (magenta) indicate colocalization. Nuclei were stained with Hoechst 33342. Scale bar 20 µm. Representative images are shown for n = 3 independent experiments. (D) Analysis of MTOR-LAMP2 colocalization by the Pearson correlation coefficient for experiment shown in (C). Data are represented as mean ± SEM, and are representative of n = 3 independent experiments. A nonparametric 2-tailed Student t test was applied; *, *P* ≤ 0.05. (E) Immunofluorescence analysis of HeLa cells that were treated as described in (C). Staining was performed with RRAGC and LAMP2 antibodies. White regions in merged image (right) of RRAGC (green) and LAMP2 (magenta) indicate colocalization. Nuclei were stained with Hoechst 33342. Scale bar 20 µm. Representative images are shown for n = 3 independent experiments. (F) Analysis of RRAGC-LAMP2 colocalization by the Pearson correlation coefficient for experiment shown in (E). Data are represented as mean ± SEM, and are representative of n = 3 independent experiments. A nonparametric 2-tailed Student *t* test was applied; ns, nonsignificant. (G) HeLa cells were either cultured in full medium or starved for amino acids and growth factors for 16 h. Immunoprecipitation (IP) was performed with PLK1 and control (mock) antibodies. Samples were analyzed by immunoblotting. Data shown are representative of n = 3 independent experiments. (H) Quantification of IP samples shown in (G). Ratio of MTOR:PLK1 was calculated for n = 3 independent experiments. Data are normalized to 1 for control condition and represented as mean ± SEM. A nonparametric 2-tailed Student *t* test was applied; *, *P* ≤ 0.05. (I) HeLa cells were treated as described in (G). IP was performed with PLK1 and control (mock) antibodies. Samples were analyzed by immunoblotting. Data shown are representative of n = 4 independent experiments. (J) Quantification of IP samples shown in (I). Ratio of RPTOR:PLK1 was calculated for n = 4 independent experiments. Data are normalized to 1 for control condition and represented as mean ± SEM. A nonparametric 2-tailed Student *t* test was applied; *, *P* ≤ 0.05.

As amino acid starvation is a condition that inhibits MTORC1 and increases autophagy, we used this condition to test if PLK1 inhibition activates MTORC1 and thereby inhibits autophagy. We first analyzed whether PLK1 contributes to MTORC1 inhibition upon amino acid starvation. To this end, HeLa cells were starved for amino acids, with or without PLK1 inhibition by short-term (30 min) BI2536 treatment. The cells were harvested at 5 to 30 min after onset of amino acid starvation (Fig. 3A). Consistent with MTORC1 inhibition, RPS6KB (p70) phospho-(T389) declined over time and was fully inhibited at 30 min after onset of amino acid starvation. Notably, RPS6KB (p70) T389 phosphorylation remained higher when PLK1 was inhibited by BI2536, as compared with the control cells (Fig. 3A, B). As the inhibitory effect of PLK1 toward MTORC1 is restricted to interphase cells, we analyzed phosphorylation of the mitotic marker H3F3 at S10 in amino acid-starved and BI2536-treated cells (Fig. S2A). H3F3 phosphorylation was high in mitotic control cells but not detectable in amino acid-starved and BI2536-treated cells, suggesting that these cultures were nonsynchronized. Thus, PLK1 inhibition led to RPS6KB (p70) T389 hyperphosphorylation in amino acid-starved interphase cells. This suggests that PLK1 restricts MTORC1 activity not only upon amino acid and insulin stimulation (Fig. 1B), but also contributes to MTORC1 inhibition in response to amino acid starvation (Fig. 3A, B).

As our data suggested that PLK1 inhibits MTORC1 by reducing its lysosomal binding (Fig. 2H, I), we next tested if PLK1 inhibition altered MTOR colocalization with the lysosomal marker LAMP2 in amino acid-starved cells. Indeed, IF analysis showed that PLK1 inhibition by BI2536 significantly increased MTOR and LAMP2 colocalization (Fig. 3C) as tested by the Pearson correlation coefficient (r _MTOR and LAMP2, control_ = 0.33, SEM = 0.02; r _MTOR and LAMP2, BI2536_ = 0.47, SEM = 0.04; P _MTOR and LAMP2, control, BI2536_ = 0.006. A nonparametric 2-tailed Student *t* test was applied.) (Fig. 3D). Localization of RRAGC/RAGC (Ras related GTP binding C), a known mediator of lysosomal MTOR localization,^14^ was not altered by BI2536 treatment (Fig. 3E,F; r _RRAGC and LAMP2, control_ = 0.53, SEM = 0.06; r _RRAGC and LAMP2, BI2536_ = 0.49, SEM = 0.04; P_ RRAGC and LAMP2, control, BI2536_ = 0.72). This is in agreement with earlier reports that RRAGC localization remains unaltered upon changes in extracellular amino acid concentrations.^14^ Thus, PLK1 inhibition aberrantly enhanced MTOR colocalization with LAMP2 in amino acid-starved cells, suggesting that PLK1 inhibits MTORC1 by decreasing its association with RRAGC-positive lysosomes.

**Figure 4.**
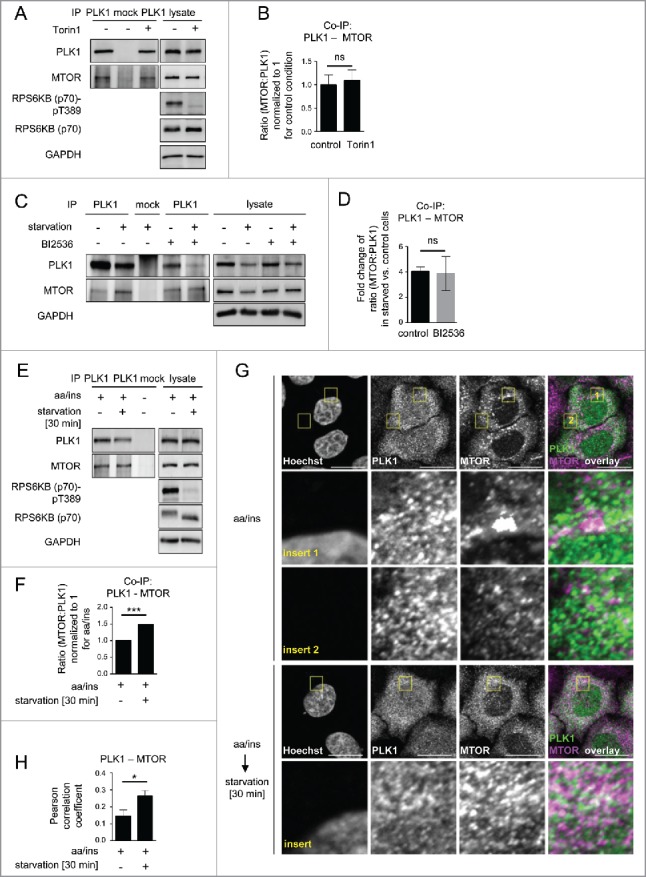
Starvation enhances PLK1-MTOR binding and cytoplasmic PLK1-MTOR colocalization. (A) HeLa cells were cultured in full medium and treated for 30 min with Torin1 or carrier, respectively. Immunoprecipitation (IP) was performed with PLK1 and control (mock) antibodies. Samples were analyzed by immunoblotting. Data shown are representative of n = 3 independent experiments. (B) Quantification of IP samples shown in (A). Ratio of MTOR:PLK1 was calculated for n = 3 independent experiments. Data are normalized to 1 for full medium condition and represented as mean ± SEM. A nonparametric 2-tailed Student *t* test was applied; ns, nonsignificant. (C) HeLa cells were either cultured in full medium or starved for amino acids and growth factors for 16 h. Cells were then treated with BI2536 or carrier, as indicated. Immunoprecipitation (IP) was performed with PLK1 and control (mock) antibodies. Samples were analyzed by immunoblotting. Data shown are representative of n = 3 independent experiments. (D) Quantification of data shown in (C). Fold change of MTOR:PLK1 ratio in starved versus control cells was calculated across n = 3 independent experiments, for carrier or BI2536 treated cells, as indicated. Data are normalized to lane 1 (C), and represented as mean ± SEM. A nonparametric 2-tailed Student *t* test was applied; ns, nonsignificant. (E) HeLa cells were starved for 1 h for amino acids and growth factors, and stimulated with amino acids and insulin for 35 min. Afterwards, for starvation, amino acids were withdrawn for 30 min. Immunoprecipitation (IP) was performed with PLK1 and control (mock) antibodies. Samples were analyzed by immunoblotting. Data shown are representative of n = 3 independent experiments. (F) Quantification of data shown in (E). Ratio of MTOR:PLK1 was calculated for n = 3 independent experiments. Data are normalized to 1 for amino acids and insulin condition, and represented as mean ± SEM. A nonparametric 2-tailed Student *t* test was applied; ***, *P* ≤ 0.001. (G) Immunofluorescence analysis of HeLa cells that were starved for 1 h for amino acids and growth factors, stimulated with amino acids and insulin for 35 min, followed by 30 min of amino acid starvation, as indicated. Staining was performed with PLK1 and MTOR antibodies. White regions in merged image (right) of PLK1 (green) and MTOR (magenta) staining indicate colocalization; insert 1 shows a region with lysosomal MTOR pattern; insert 2 shows a cytoplasmic region without lysosomal MTOR pattern. Nuclei were stained with Hoechst 33342. Scale bar: 20 µm. Representative images are shown for n = 3 independent experiments. (H) Analysis of PLK1-MTOR colocalization by the Pearson correlation coefficient for experiment shown in (G). Ten representative cells were quantified for each condition. Data are represented as mean ± SEM and are representative of n = 3 independent experiments. A nonparametric 2-tailed Student *t* test was applied; *, *P* ≤ 0.05. (E, F, G, H) aa, amino acids; ins, insulin.

As our earlier data indicated that MYC-PLK1 (WT) overexpression inhibits MTORC1 (Fig. 2I to J), and the extent of interaction between them may contribute to PLK1-mediated MTORC1 inhibition, we next tested whether endogenous PLK1-MTORC1 binding was altered by amino acid starvation. Therefore, we performed PLK1 IPs from amino acid-starved or full medium-cultivated cells. We found that the signals for both MTOR (Fig. 3G, H) and RPTOR (Fig. 3I, J) were increased in PLK1 IPs from amino acid-starved cells. We consistently immunoprecipitated less PLK1 from amino acid-starved cells, which led to a decrease in PLK1 signals (Fig. 3G, I). Nevertheless, the signals for coimmunoprecipitated MTOR and RPTOR were stronger for PLK1 IPs from amino acid-starved cells as compared with full medium-cultivated cells (Fig. 3G, I), indicating an increase in PLK1-MTOR and -RPTOR binding under amino acid starvation. To quantify the relative amount of RPTOR or MTOR bound to PLK1 in nonstarved versus amino acid-starved cells, we normalized the RPTOR and MTOR signals to the PLK1 levels in each respective IP. We found that physical PLK1 interaction with RPTOR and MTOR significantly increased upon amino acid withdrawal (Fig. 3H, J). We conclude that increased MTORC1-PLK1 binding occurs when MTORC1 is inhibited by amino acid starvation. This is consistent with our earlier finding that overexpression of active PLK1 led to increased PLK1-MTORC1 binding and reduced lysosomal association of the PLK1-MTORC1 complex, correlating with reduced MTORC1 activity (Fig. 2H to J). As amino acid deprivation inhibits MTORC1, we tested if MTOR inhibition by Torin1 could phenocopy the observed increase in PLK1-MTOR binding in amino acid-starved cells (Fig. 4A, B). Therefore, we performed MTOR IPs from HeLa cells cultivated in full medium and treated for 30 min with Torin1 or carrier (Fig. 4A, B). Torin1 inhibited RPS6KB (p70) T389 phosphorylation but did not alter PLK1-MTOR binding, suggesting that MTORC1 kinase activity does not control its own binding to PLK1. Next we tested if PLK1 activity affected the induction of PLK1-MTOR binding by amino acid starvation. Therefore, we performed PLK1 IPs from HeLa cells that were treated with the PLK1 inhibitor BI2536 or carrier, and starved for amino acids or cultivated in full medium. Amino acid withdrawal enhanced endogenous PLK1-MTOR binding 4-fold to the same extent in the presence or absence of BI2536 (Fig. 4C, D), suggesting that PLK1 kinase activity does not mediate enhanced PLK1-MTOR binding upon amino acid deprivation. Thus, amino acid deprivation may represent an input that is separate from MTORC1 and PLK1, as inhibition of MTOR or PLK1 did not alter increased PLK1-MTOR binding in amino acid-starved cells. Of note, we observed that acute amino acid starvation not only significantly enhanced PLK1-MTOR binding (Fig. 4E, F) but also cytoplasmic colocalization of PLK1 and MTOR (Fig. 4G, H). This suggests that enhanced PLK1-MTOR association in amino acid-deprived cells may contribute to MTORC1 inhibition, via PLK1-mediated MTORC1 localization away from lysosomes.

As MTORC1 inhibition derepresses autophagy,^10^ we next tested if PLK1 via MTORC1 inhibition enhances autophagy. To this end, we inhibited PLK1 by BI2536 in amino acid-starved and control cells and detected MAP1LC3A/LC3 (microtubule-associated protein 1 light chain 3 α)^35^ which is a widely used autophagy marker.^35,60^ Unprocessed LC3 (LC3-I) is soluble and resides in the cytoplasm. Upon autophagy induction, LC3-I is processed at its C terminus and conjugated to phoshatidylethanolamine (referred to as LC3-II). LC3-II associates with autophagosomal inner and outer membranes,^60^ and becomes degraded upon fusion with lysosomes.^28,30^ Yet, dual processing of LC3 renders the interpretation of LC3-II signals challenging.^35^ On the one hand, LC3 is lipidated and integrated into the phagophore membrane (the precursor to the autophagosome), leading to an increase in LC3-II signal in immunoblots. On the other hand, LC3-II is degraded by lysosomal proteases upon autophagosomal-lysosomal fusion, decreasing the LC3-II signal. Thus, LC3-II degradation can mask the increase in LC3-II upon autophagy induction. To prevent LC3-II degradation and enable detection of LC3-II accumulation, we supplemented all media for autophagy assays with the v-ATPase inhibitor bafilomycin A_1_ (BafA). BafA inhibits autophagosomal-lysosomal fusion, a late step in the autophagy process. Thus, LC3-II can still be integrated into the phagophore membrane, but it is no longer degraded by lysosomal proteases, and LC3-II accumulation can be reliably detected. In keeping with this, BafA strongly induced LC3-II levels in HeLa cells (Fig. S2D).

**Figure 5.**
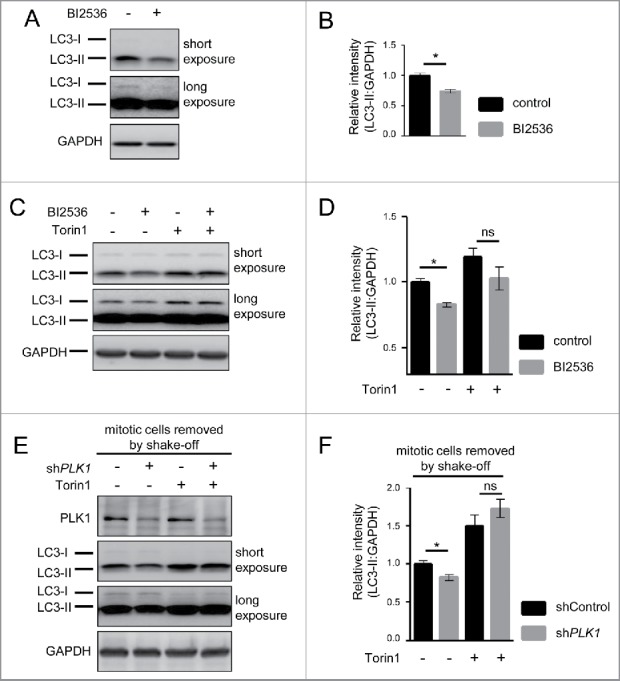
PLK1 inhibition reduces the autophagy marker LC3-II in interphase cells. (A) HeLa cells were starved for 1 h for amino acids and growth factors, stimulated with amino acids and insulin for 35 min, followed by 30 min amino acid starvation. All media were supplemented with bafilomycin A_1_. BI2536 was added as indicated for 30 min. Data shown are representative of n = 3 independent experiments. (B) Quantification of data shown in (A). Ratio of LC3-II:GAPDH was calculated for n = 3 independent experiments. Data are normalized to 1 for the control condition and represented as mean ± SEM. A nonparametric 2-tailed Student *t* test was applied; *, *P* ≤ 0.05. (C) HeLa cells were treated with BI2536 and/or Torin1 as indicated, and stimulated as described in (A). Samples were analyzed by immunoblotting. Data shown are representative of n = 3 independent experiments. (D) Quantification of data shown in (C). Ratio of LC3-II:GAPDH was calculated for n = 3 independent experiments. Data are normalized to 1 for the control condition (no Torin1, no BI2536) and represented as mean ± SEM. A nonparametric 2-tailed Student *t* test was applied; ns, nonsignificant; *, *P* ≤ 0.05. (E) *PLK1* (sh*PLK1*) or control (shControl) shRNA HeLa cells were starved for 1 h for amino acids and growth factors, stimulated with amino acids and insulin for 35 min, followed by 20 min amino acid starvation. All media were supplemented with bafilomycin A_1_. Cells were treated with Torin1 as indicated. Mitotic cells were removed by shake-off. Hence, only interphase cells were analyzed. Data shown are representative of n = 4 independent experiments. (F) Quantification of data shown in (E). Ratio of LC3-II:GAPDH was calculated for n = 4 independent experiments. Data are normalized to 1 for the shControl condition (no Torin1) and represented as mean ± SEM. A nonparametric 2-tailed Student *t* test was applied; ns, nonsignificant; *, *P* ≤ 0.05.

Upon amino acid starvation for 30 min, we observed that PLK1 inhibition by BI2536 caused a significant decrease in LC3-II levels (Fig. 5A, B), indicating that PLK1 plays a positive role in autophagy. Next, we tested whether LC3-II reduction by PLK1 inhibition required MTOR activity. To this end, we combined PLK1 inhibition by BI2536 with MTOR inhibition by Torin1, and starved cells for amino acids (Fig. 5C, D). Whereas PLK1 inhibition by BI2536 significantly reduced LC3-II levels, BI2536 had no significant effect on LC3-II levels when combined with the MTOR inhibitor Torin1 (Fig. 5C, D). We also analyzed LC3-II levels in shControl and sh*PLK1* knockdown cells, without and with Torin1 treatment. In these experiments, mitotic cells were removed by shake off. PLK1 knockdown significantly reduced LC3-II, and this effect was suppressed by Torin1 treatment (Fig. 5E, F). Thus, LC3-II reduction by PLK1 inhibition or knockdown required MTOR activity, suggesting that PLK1 positively contributes to autophagy by inhibiting MTORC1.

**Figure 6.**
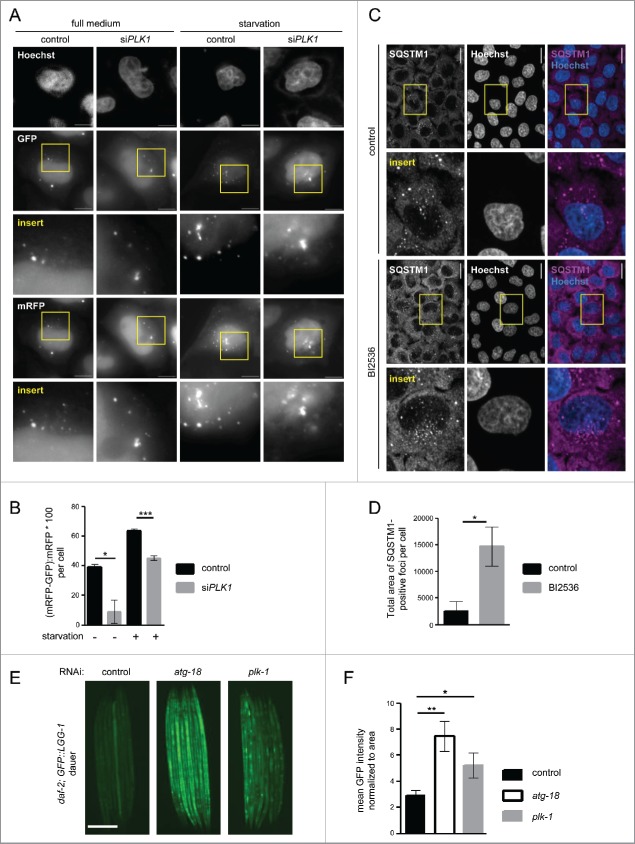
PLK1 inhibition impairs autophagy in nonmitotic cells and in *C. elegans* dauers. (A) HeLa cells were transfected with mRFP-GFP-LC3 tandem reporter, followed by *PLK1* siRNA transfection on the next day. Cells were either kept in full medium, or starved for serum and amino acids for 16 h. Mitotic cells were removed by shake-off before fixation of cells 24 h after siRNA transfection. Representative images are shown for each condition. Scale bar 10 µm. Data shown are representative of n = 2 independent experiments. (B) Quantification of experiment shown in (A). The numbers of green puncta (autophagosomes) and red puncta (autolysosomes plus autophagosomes) were counted for nonmitotic cells. Data shown are represented as mean ± SEM for n = 30 cells for control knockdown, full medium, n = 43 cells for si*PLK1*, full medium, n = 35 cells for control knockdown, starvation condition, and n = 26 for si*PLK1* starvation condition. A nonparametric 2-tailed Student *t* test was applied; *, *P* ≤ 0.05; ***, *P* ≤ 0.001. (C) Immunofluorescence analysis of HeLa cells that were starved for 1 h for amino acids and growth factors, stimulated with amino acids and insulin for 35 min, followed by 30 min amino acid starvation. All media were supplemented with bafilomycin A_1_. Staining was performed with SQSTM1 antibody and Hoechst 33342. Shown are maximum intensity projections. Scale bar 20 µm. Representative images are shown for n = 3 independent experiments. (D) Quantification of experiment shown in (C). The total area of SQSTM1-positive foci was calculated and normalized to the number of nuclei. n = 126 cells for control condition and n = 105 cells for BI2536 treated condition. Data are represented as mean ± SEM, and are representative of n = 3 independent experiments. A nonparametric 2-tailed Student *t* test was applied; *, *P* ≤ 0.05. (E& F) *daf-2(e1370)* animals expressing GFP::LGG-1 were fed bacteria expressing control, *atg-18* or *plk-1* dsRNA from hatching, and raised at the nonpermissible temperature (25°C) to induce dauers. (E) Micrographs of ∼8 to 10 dauer animals lined up next to each other were taken 6 d after the temperature shift. Scale bar 200 µm. (F) GFP::LGG-1 fluorescence (mean ± s.d. of ∼8–10 animals, **P<0.0001, one-way ANOVA) was quantified. Data shown are representative of 3 independent experiments.

To further validate a role for PLK1 in autophagy regulation, we used a tandem mRFP-GFP-LC3 reporter,^35,60,61^ which is a standard tool to monitor the status of the autophagy process. GFP (green fluorescent protein) displays higher sensitivity to low pH than mRFP^61^ (monomeric red fluorescent protein). Therefore, the tandem mRFP-GFP-LC3 reporter allows tracking of acidification of autolysosomes by providing a readout for autophagosome and autolysosome numbers.^35,60,61^ HeLa cells were transiently transfected with the reporter construct in combination with PLK1 or control siRNA knockdown, and subjected to full-medium conditions or amino acid starvation. Mitotic cells were removed by shake-off. Fixed cells were stained with Hoechst, imaged by wide-field microscopy (Fig. 6A), and deconvoluted images were analyzed as described previously.^62^ The few remaining mitotic cells, as determined by chromatin condensation state detected by Hoechst DNA staining, were omitted from the analysis. GFP puncta, representing autophagosomes, and mRFP puncta, representing autolysosomes plus autophagosomes, were counted. To determine the percentage of autolysosomes, we subsequently calculated the difference between mRFP and GFP puncta, which we expressed as the percentage of all mRFP positive puncta per cell (Fig. 6B). As expected, starvation increased the percentage of autolysosomes consistent with enhanced autophagy. PLK1 knockdown reduced the percentage of autolysosomes under full medium conditions and upon amino acid starvation (Fig. 6B). This is in agreement with the decline in LC3-II levels upon PLK1 inhibition detected by immunoblotting (Fig. 5A to F).

We further consolidated this finding by analyzing the autophagy substrate SQSTM1/p62 (sequestosome 1). SQSTM1 is recruited by LC3 into autophagosomes, and thus SQSTM1 punctate structures represent LC3-positive autophagosome-associating SQSTM1. When autophagy is blocked, SQSTM1 foci accumulate due to inefficient autophagosome turnover.^35^ We detected SQSTM1 foci by IF in amino acid-starved cells that were treated with the PLK1 inhibitor BI2536 or carrier (Fig. 6C, D). In agreement with the reduced LC3-II levels (Fig. 5A to F) and decreased percentage of autolysosomes (Fig. 6B), we found that SQSTM1 foci numbers were significantly increased in PLK1 inhibited cells, compared with the control cells, thus providing further evidence that PLK1 is a positive regulator of autophagy.

The autophagy analyses reported above were performed in interphase cells. However, we could not rule out that PLK1 may regulate autophagy in mitotic cells as well. To test this, we analyzed LC3-II during mitosis. We arrested cells in mitosis by a consecutive aphidicolin-nocodazole block, released them for different times as indicated (Fig. S2E), and detected cell cycle markers and LC3-II by immunoblotting. Consistent with an increased amount of mitotic cells in the culture, we observed increased phosphorylation of H3F3 at serine 10 and decreased levels of the G1/S phase marker CCNE1. Autophagy, as monitored by LC3-II levels, was low in mitotic cells as compared with control cells from an asynchronous culture (Fig. S2E), which is consistent with previous reports.^36,37^ Thus, we conclude that mitotic cells display low autophagy, suggesting that PLK1 may promote autophagy primarily in nonmitotic cells. This is also in agreement with our finding that autophagic control by PLK1 depends on MTOR activity (Fig. 5C to F), which is inhibited during mitosis (Fig. 1F).

We next aimed to determine whether PLK1s role in autophagy in nonmitotic cells is conserved from invertebrates to mammals. For this purpose, we used the genetic model organism *C. elegans*. We analyzed the role of *plk-1*, the *C. elegans* PLK1 ortholog, in dauer larvae, a developmentally arrested stage of *C. elegans* in which the animals display cell cycle quiescence and therefore consist of nonmitotic cells. Dauer entry and G_1_ cell cycle arrest in *C. elegans* larvae occur in response to environmental stresses, including starvation.^63^ Specifically, we used animals carrying a thermosensitive, mutant allele for the INSR-IGF1R homolog DAF-2, *daf-2(e1370)*, and stably expressing the transgene GFP::LGG-1^64^ (orthologous to mammalian LC3; scheme of the experimental setup provided in Fig. S2F). *daf-2(e1370)* mutants enter the dauer stage upon shift to the restrictive temperature (25°C), during which markers of autophagy are increased.^65^ Moreover, RNAi knockdown of genes involved in the autophagic process changes the subcellular localization of GFP::LGG-1 in hypodermal cells of *daf-2(e1370)* mutants, while causing an enhanced GFP::LGG-1 signal in these cells.^65^ Consistent with these observations, we found a 3-fold increase in GFP::LGG-1 intensity in the body of *daf-2(e1370)* dauers subjected to RNAi-mediated inhibition of the autophagy WIPI protein *atg-18*,^66^ compared with control RNAi (Fig. 6E, F). This indicates that inhibition of autophagy causes increased GFP::LGG-1 levels in *daf-2* dauer larvae. When we quantified GFP::LGG-1 intensity in *daf-2* dauers subjected to *plk-1* RNAi, we observed that inhibition of *plk-1*, like inhibition of *atg-18*, significantly increased GFP::LGG-1 levels compared with dauers subjected to control RNAi (Fig. 6E, F). Thus, *plk-1* RNAi, similar to inhibition of PLK1 in mammalian cells, appeared to cause a block of autophagy, suggesting that PLK1/PLK-1 is a conserved regulator of autophagy. As *C. elegans* dauer larvae consist of G_1_/S interphase cells,^63^ our data further suggest that, similarly to mammalian cells, *C. elegans* PLK-1 positively regulates autophagy in nonmitotic cells.

## Discussion

In the present study, we show that PLK1 physically binds and phosphorylates MTORC1. In interphase cells, inhibition of PLK1 increases MTORC1 activity, as measured by RPS6KB (p70) phosphorylation at T389. Consistently, MTORC1′s lysosomal association (Fig. 2I) and activity (Fig. 2J) are decreased in cells overexpressing active PLK1, as compared with the inactive protein. In line with this, PLK1 inhibition mitigates autophagy in an MTOR-dependent manner (Fig. 5C to F). Positive regulation of autophagy by PLK1/PLK-1 occurs in *C. elegans* and mammalian cells, suggesting that this PLK1 function is evolutionary conserved.

PLK1 is mainly perceived as a regulator of mitotic progression.^2,3^ Here we describe a novel function of PLK1 in interphase cells where it inhibits MTORC1 and activates autophagy under nutrient sufficiency and amino acid deprivation. Our data suggest that the functions of PLK1 in mitotic and interphase cells are mediated by distinct mechanisms since *(i)* PLK1 inhibition increases MTORC1 activity in interphase cells (Fig. 1G, H) but not in mitotic cells (Fig. 1D, E); *(ii)* PLK1 inhibition reduces autophagy in interphase cells (Figs. 5 and 6). In contrast, mitotic cells display high PLK1 levels^46,47^ (Fig. 1F) but low autophagy (Fig. S2E), suggesting that PLK1 promotes autophagy primarily in nonmitotic cells; (*iii*) autophagy inhibition by PLK1 inhibition depends on MTOR activity (Fig. 5C to F). MTORC1 in mitotic cells is inhibited (Fig. 1F) and cannot be activated by PLK1 inhibition (Fig. 1D, E). Thus, we conclude that PLK1 inhibits MTORC1 and activates autophagy in interphase but not in mitotic cells. Which mechanisms may coordinate PLK1's mitotic and interphase functions is currently unknown and will require further investigation.

Our finding that PLK1 inhibits MTORC1 in interphase but not in mitotic cells helps to resolve seemingly contradictory reports on the effects of PLK1 inhibition on MTORC1. For example, our findings are in agreement with Spartà et al.^26^ who report that the PLK1 inhibitor BI6727 increases RPS6KB (p70) and RPS6 phosphorylation. Yet, this has been so far debatable as 4 other studies^22-25^ reported that PLK1 inhibition suppresses the phosphorylation of MTORC1 substrates. At first glance this seems to be at odds with our findings and those of Spartà et al.^26^ However, Renner et al.^22^ Astrinidis et al.,^23^ Zhang et al.^24^ and Li et al.^25^ use long-term treatments with PLK1 inhibitors or siRNA, increasing the amounts of mitotic cells in the cultures. In some studies, long-term PLK1 inhibition is even combined with a mitotic block.^22,23^ Thus, the reduced MTORC1 activity reported in those studies is measured in mitotic cells. In agreement with those data, we also show that MTORC1 is inhibited in mitotic cells (Fig. 1F). However, after removal of mitotic cells, our data reveal that PLK1 inhibition activates MTORC1 in interphase cells (Fig. 1G), which corresponds with data from Spartà et al.^26^ Thus, our findings resolve and unify earlier—seemingly paradoxical—findings on PLK1 inhibitor effects on the MTORC1 substrate RPS6KB (p70).

Our results also complement previous studies on PLK1 inhibitor effects on autophagy. We observed here that PLK1 inhibition causes a decline in autophagy in interphase cells, as determined by the reduction in LC3-II accumulation and autolysosome numbers (Fig. 5, Fig. 6A, 6B). In agreement, Valianou et al.^39^ show in TSC1- or TSC2-deficient lymphangioleiomyomatosis patient derived cells that BI2536 moderately inhibits autophagy. Our finding that PLK1 regulates MTORC1 adds to the interpretation of these data. As loss of TSC1 or TSC2 leads to massive MTORC1 hyperactivation, MTORC1 can most probably not be much further activated by PLK1 inhibition in a TSC1- or TSC2-deficient background. This may explain the only moderate effect of BI2536 on autophagy observed in that study. Another study in LNCaP cells reports that long-term treatment with BI2536 for 5 d leads to mitotic arrest and necroptosis, correlating with cell death related autophagy activation.^40^ In our hands, autophagy was decreased in HeLa cells upon a 38 h mitotic block (Fig. S2E). Thus, autophagic activity during mitosis may vary depending on the length of cell cycle arrest and the cell type studied.

We find here that the MTORC1 component RPTOR is directly phosphorylated *in vitro* by PLK1. Which RPTOR residues may be PLK1 substrate sites? We analyzed the RPTOR sequence for known consensus phosphorylation motifs of PLK1,^67^ but did not find any. Thus, PLK1 substrate sites in RPTOR cannot be theoretically predicted. Three RPTOR phosphorylation sites at S722, S863, and S877 have been previously identified by 2 studies,^68,69^ which report on PLK1-dependent mitotic phosphoproteomes. We did not observe changes in phosphorylation of RPTOR at S722 and S863 upon BI2536 treatment in nonmitotic cells (Fig. S2G). For RPTOR phospho-(S877), we did not detect a specific signal with the available antibody (data not shown). Also other reported RPTOR phosphorylation sites^70^ at S859 and T706 remained unchanged by BI2536 (Fig. S2G). Thus, further studies are needed to gain insight into RPTOR residues that are phosphorylated by PLK1 in interphase cells. Likewise, discovery proteomic studies are required to identify other interphase substrates and thereby more generally delineate the interphase response of the PLK1 phosphoproteome to changes in nutrient supply.

The central platform for MTORC1 signaling is the lysosome, which is also the essential compartment for autophagy. Consistent with a role for PLK1 in MTORC1 regulation and autophagy, we report that PLK1 colocalizes with MTOR in a lysosomal pattern (Fig. 2A, B) and the lysosomal marker LAMP2 coimmunoprecipitates with PLK1 (Fig. 2G). Furthermore, in sucrose gradients PLK1 is detected in the lysosomal fraction, jointly with MTOR and RPTOR, and the MTORC1 regulator TSC2 (Fig. 2E, F). This finding is intriguing as there is so far no other report on lysosomal targeting of PLK1. Consistent with PLK1s lysosomal association reported here, PLK1 contains a GY motif which is a lysosomal targeting signal.^71^

Under which physiological conditions and in response to which stimuli does PLK1 inhibit MTORC1? We find here that PLK1 inhibition increases MTORC1 activity both under nutrient sufficiency (Fig. 1B, C, G to J) and amino acid withdrawal (Fig. 3A, B), and the extent of induction of RPS6KB (p70) T389 phosphorylation by PLK1 inhibition is equally strong in nutrient-induced (Fig. 1C, H) and starved cells (Fig. 3B). This suggests that PLK1 is active and inhibits MTORC1 in both conditions. Of note, short- and long-term starvation enhances binding of endogenous PLK1 with MTORC1 (Fig. 3G to J, 4E, F), and this is independent of MTORC1 or PLK1 kinase activity (Fig. 4A to D). This suggests that amino acid starvation regulates upstream cues, which cause enhanced PLK1-MTOR association. The molecular mediators that control starvation-induced PLK1-MTOR binding remain to be determined. Whereas PLK1 kinase activity does not affect starvation-induced PLK1-MTOR binding (Fig. 4C, D), PLK1 inhibition does lead to aberrant lysosomal localization of the PLK1-MTOR complex (Fig. 3C, D). This suggests that the increased PLK1-MTOR interaction in response to amino acid starvation does not happen at the lysosome, but another localization, e.g., in the cytoplasm. This is in agreement with the enhanced cytoplasmic colocalization of MTOR and PLK1 upon amino acid starvation (Fig. 4G, H). This implies that enhanced PLK1-MTOR binding in starved cells is a separate mechanism that indirectly adds to PLK1s inhibitory effect on MTORC1 via localization away from lysosomes (scheme on the 2 separate mechanisms provided in Fig. S2H).

Similar to MTORC1 activity, its localization as well as autophagy are altered by PLK1 inhibition both under nutrient starvation and sufficiency. Increased lysosomal localization or binding of MTOR upon PLK1 inactivation can be detected in starved (Fig. 3C, D) and full medium cultivated cells (Fig. 2I). Hence, PLK1 contributes under both conditions to MTOR relocalization away from lysosomes. In keeping with this, PLK1 inhibition mitigates autophagy both under full medium conditions and in starved cells (Fig. 6A, B). We conclude that MTORC1 inhibition by PLK1 is a general mechanism, which is not restricted to starved cells only, and consequently PLK1 positively contributes to autophagy both under starvation and nutrient-replete conditions (see scheme Fig. S2H). Of note, autophagy does not only occur in starved cells, but is also a critical housekeeping and prosurvival pathway under nutrient sufficiency. Basal autophagy maintains, for example, protein homeostasis by removing misfolded proteins, and mobilizes cellular energy and nutrient stores to maintain a stable pool of metabolite intermediates (reviewed by Kaur et al^29^).

Our finding that PLK1, next to mitotic progression, promotes autophagy in interphase cells suggests that for therapies of low grade tumors, which typically contain only 5% to 10% mitotic cells, PLK1 inhibitors may perform better than other purely antimitotic agents. As novel therapeutics are often tested first in advanced tumors, this point may have been missed so far, and clinical studies are needed to address performance of PLK1 inhibitors vs other antimitotics such as paclitaxel in low grade tumors. Beyond this, our findings suggest that combinatorial targeting of MTORC1 and PLK1 may hold promise for cancer treatment. PLK1^1,4,72^ and MTOR^8,9^ are common drug targets in cancer therapy, but combinatorial treatments are rarely tested even in preclinical studies. It seems promising that combination of the dual phosphoinositide 3-kinase-MTOR inhibitor BEZ235 and the PLK1 inhibitor BI2536 in xenograft models of colorectal cancer shows that either inhibitor alone fails to enhance apoptosis, but combinatorial treatment inhibits MTORC1 readouts and leads to massive tumor cell death.^73^ We show here that PLK1 inhibition can activate MTORC1 and suppress autophagy. As this may affect tumor cell survival and growth, we advocate cautious monitoring of MTORC1 and autophagy readouts in clinical trials with PLK1 inhibitors. Correlation of such data with clinical outcome may allow development of strategies for optimized (combinatorial) cancer therapies, to simultaneously target PLK1 and MTOR in tumors where MTORC1 is activated by PLK1 inhibition.

## Materials and methods

### Nomenclature

Genes and proteins are designated according to the recommendations of the HUGO gene nomenclature committee (HGNC).

### Cell culture and cell treatments

HeLa α Kyoto cells were cultivated in full medium Dulbecco's modified Eagle's medium (DMEM; PAN Biotech, P04–03600) supplemented with 10% fetal calf serum (FCS; PAA, A15–102, Lot A10208–0991), 3 mM L-glutamine (Gibco, Life Technologies, 25030–024) at 37°C, 7.5% CO_2_. For stimulation with amino acids and insulin, cells were cultivated in DMEM, supplemented with 3 mM L-glutamine and 100 nM insulin (Sigma-Aldrich, I1882), for the indicated time points. Prior to starvation experiments, cells were washed twice with phosphate-buffered saline (PBS; PAN Biotech, P04–36500). Starvation was either performed for amino acids and growth factors in Hank's balanced salt solution (HBSS; PAN Biotech, P04–32505), or for amino acids in amino acid-free DMEM (PAN Biotech, P04–01507) supplemented with 4.5 g/l glucose (Sigma-Aldrich, G7021) and 100 nM insulin, as indicated. Mitotic shake-off was performed where indicated to remove the mitotic cells. Prior to the mitotic shake-off, cells were starved 16 h for amino acids and growth factors in HBSS. Nocodazole or consecutive aphidicolin-nocodazole treatment were performed as described before.^74^ In brief, for consecutive aphidicolin-nocodazole treatment cells were treated 16 h with 1.6 μg/mL aphidicolin (Sigma-Aldrich, A0781), followed by a release into the cell cycle using full medium for 7 h and subsequently treated with 100 ng/mL nocodazole (Sigma-Aldrich, M1404) for 15 h, followed by release for the indicated times.

siRNA knockdown of *PLK1* was induced using ON-TARGET plus SMARTpool siRNA, final concentration 10 nM (Dharmacon, L-003290–00). siRNA transfection was performed using Lipofectamine 2000, (Life Technologies, 11668–019) and DNA transfection was performed with JetPEI (PolyPlus, 101–40) according to the manufacturer's protocol.

Overexpression of PLK1 was performed using the following constructs: empty vector pRcCMV (Invitrogen V75020), pRcCMV MYC-PLK1^K82R^ (Addgene plasmid 41157, deposited by Erich Nigg),^59^ and pRcCMV MYC-PLK1 (WT) (Addgene plasmid 41160, deposited by Erich Nigg).^38^ The medium was exchanged 6 h post transfection. Cells were harvested after removal of the mitotic cells by mitotic shake-off 24 h or 48 h post transfection, with similar results.

The sh*PLK1* HeLa cell line was generated using the pTRIPZ system (Dharmacon). For virus generation HEK293T cells were cotransfected using jetPEI with the *PLK1* shRNA construct, (target sequence sh*PLK1*: CTGTCTGAAGCATCTTCTG; Dharmacon, RHS4740-EG5347) or a nontargeting control sequence, respectively, with the Trans-Lentiviral shRNA Packaging system. The virus supernatant was harvested 72 h after transfection. HeLa cells were seeded in the morning and the infection with the virus supernatant was performed for 16 h. The transduction step was repeated twice. Selection of successfully transduced cells was achieved by adding puromycin (final concentration 2 µg/mL; Sigma-Aldrich, P8833). A stably transduced doxycycline-inducible HeLa sh*RPTOR* cell line and a control cell line (shControl) harboring a nontargeting control sequence were described previously.^45^ Knockdown was induced with doxycyline for 3 d (final concentration 2 µg/mL; Sigma-Aldrich, D3447). Doxycycline was removed for 16 h before the start of all experiments in this study.

### Antibodies and reagents

The following antibodies were purchased from Cell Signaling Technology, Inc.: RPTOR (2280), MTOR (2983), RPS6KB (p70) phospho-(T389) (9206), RPS6KB (p70) (9202), LC3 (2775), ULK1-phospho-(S757) (6888), ULK1 (4773), LMNA (2032), CCNE1 (4129). GAPDH antibody was bought at Abcam (ab8245). PLK1 (sc-55504), LAMP2 (sc-18822), RPTOR phospho-(S863) (sc-130214), and MYC/c-Myc (sc-40) antibodies for immunoblotting and normal mouse IgG (sc-2025) and normal rat IgG (sc-2026) for immunoprecipitation were obtained from Santa Cruz Biotechnology, Inc. H3F3/histone H3 phospho-(S10) (A301–844A) and H3F3/histone H3 (A300–822A) antibodies were bought from Bethyl Laboratories, Inc. The HA antibody (11867423001) was obtained from Roche. ACTIN (MAB1501) and RPTOR phospho-(S722) (09–104) antibodies were purchased from Merck Millipore. RPTOR phospho-(T706), RPTOR phospho-(S859) and RPTOR phospho-(S877)^70^ were a kind gift from Diane C. Fingar (University of Michigan Medical School, Ann Arbor, MI, USA). Horseradish peroxidase-conjugated goat anti-mouse (31430) and goat anti-rabbit IgG (31460) were ordered from Thermo Scientific Pierce, horseradish peroxidase-conjugated light chain specific antibody for blotting after IP was obtained from Jackson ImmunoReseach Laboratories, Inc. (115–035–174). For immunofluorescence experiments, the RRAGC/RAGC (9480) and MTOR (2983) antibodies were purchased from Cell Signaling Technology, Inc., LAMP2 (sc-18822) and PLK1 (sc-17783) were bought from Santa Cruz, Biotechnology, Inc. The SQSTM1/p62 antibody was obtained from Progen Biotechnik (GP62-C). All secondary antibodies for immunofluorescence experiments were bought from Thermo Fisher Scientific: Goat anti-guinea pig IgG (H+L), Alexa Fluor® 568 conjugate (A-11075), goat anti-rabbit IgG (H+L), Alexa Fluor® 568 conjugate (A-11036), goat anti-rabbit IgG (H+L), Alexa Fluor® 488 conjugate (A-11008), goat anti-mouse IgG (H+L), Alexa Fluor® 488 conjugate (A-11001), and goat anti-mouse IgG (H+L), Alexa Fluor® 568 (A-11004). Bafilomycin A_1_ was bought at Tebu-Bio (BIA-B1012) and throughout the study used at a final concentration of 100 nM. PLK1 inhibitor BI2536 (Axon Medchem, 1129) was used at 100 nM final concentration and added 30 min before lysis throughout the study unless otherwise stated and MTOR inhibitor Torin1 (Axon MedChem, 1833), was used at 250 nM and added 30 min before stimulation with amino acids and insulin.

### Cell lysis and immunoblotting

HeLa cells were washed twice with PBS before lysis in RIPA lysis buffer (1% NP40 [Sigma-Aldrich, I8896], 0.1% sodium dodecyl sulfate [Sigma-Aldrich, 71725], 0.5% sodium deoxycholate [Sigma-Aldrich, 30970] in PBS) supplemented with Complete Protease Inhibitor Cocktail (Roche, 11836145001), Phosphatase Inhibitor Cocktail 2 (Sigma-Aldrich, P5726) and Cocktail 3 (Sigma-Aldrich, P0044).

Adjustment of the protein concentration, SDS PAGE and immunoblot were performed as described previously.^45^ Pierce ECL western blotting substrate (32209) or SuperSignal West FEMTO (34095), both from Thermo Scientific Pierce, were used to detect chemiluminescence using a LAS-4000 mini camera system (Fujifilm Life Science Systems, Tokyo, JP) or a LAS-4000 mini camera system (GE Healthcare, Little Chalfont, UK).

Quantification of raw image files was performed using ImageQuant TL Version 8.1, GE Healthcare. Background subtraction was performed using the rolling ball method with a defined radius of 200 for all images.

For graphical representation, raw images from the Fujifilm camera were exported as Color TIFF files using the Fujifilm software Multi Gauge version 3.0, Fujifilm Life Science Systems, and further processed with Adobe Photoshop version CS2. Raw images taken with the LAS-4000 mini, GE Healthcare system were exported as RGB color TIFF files using ImageJ, and further processed with Adobe Photoshop version CS5.1.

### Immunoprecipitation (IP)

HeLa cells were washed 3x with ice-cold PBS and harvested in IP lysis buffer (40 mM HEPES, 120 mM NaCl and 0.3% CHAPS [Sigma-Aldrich, 000000010810118001], pH 7.5) supplemented with Complete (Roche, 11836145001), Phosphatase Inhibitor Cocktail 2 and Cocktail 3 (Sigma-Aldrich, P5726, P0044). Lysates were precleaned by adding 10 µL/mL magnetic Dynabeads® Protein G (Life Technologies, 10009D), prewashed in lysis buffer, for 30 min at 4°C with end-over-end rotation. A lysate sample was taken up in 5x SDS sample buffer (50% glycerol, 5% β-mercaptoethanol, 0.3 M SDS, 0.03 M Tris, pH 6.8, 0.2 µM bromophenol blue) for subsequent analysis by immunoblot. IP was performed by adding 7.5 µg of a specific antibody or control IgG (“mock,” negative control) per mL lysate for 30 min at 4°C. Subsequently, 37.5 µL prewashed Dynabeads® Protein G per mL lysate were added to the IP reactions for 1.5 h at 4°C. Beads were washed 3 times briefly and twice for 10 min in IP lysis buffer and resuspended in 1x SDS sample buffer.

### PLK1 kinase assay

HeLa cells were transfected with pRK5-HA-RPTOR (Addgene plasmid 8513,^52^ gift from David Sabatini) 48 h before the experiment. HA-RPTOR was immunopurified using an HA antibody. A control (mock) IP was performed with rat IgG. The immunoprecipitates were dephosphorylated with alkaline phosphatase (10 U; Thermo Scientific, EF0652) for 1 h at 37°C, and washed with IP lysis buffer, followed by a washing step with kinase assay buffer (20 mM HEPES, pH 7,4, 150 mM KCl, 10 mM MgCl_2_). Recombinant PLK1 (0.1 µg; Enzo Life Sciences, BML-SE466–0005) was added to RPTOR and mock IPs as indicated. The kinase-substrate mixture was preincubated on ice for 15 min with BI2536 (100 nM), Torin1 (250 nM) or carrier, respectively. An ATP mix with 1 mM cold ATP (GE Healthcare, 27–1006–01) and 5 to 10 µCi [γ-33P] ATP (PerkinElmer, NEG302H250UC) was added and incubated for 30 min at 30°C with gentle shaking. Samples were washed once with kinase assay buffer before resuspension in 1x SDS sample buffer and heated for 15 min at 68°C. Samples were separated by SDS-PAGE and phosphorylation was analyzed by autoradiography. For quantification the signal that was measured for the condition without PLK1 was considered as background and thus subtracted. For nonradioactive assay the same protocol was performed, 0.4 µM cold ATP was added and samples were analyzed by immunoblotting.

### Sucrose gradient

HeLa cells were treated as indicated and lysed in homogenization buffer (50 mM Tris-HCl, pH 7.4, 250 mM sucrose [Sigma-Aldrich, S2395], 25 mM KCl, 5 mM MgCl_2_, 3 mM imidazole), supplemented with Complete (Roche, 11836145001) and Phosphatase Inhibitor Cocktail 2 and Cocktail 3 (Sigma-Aldrich, P5726, P0044). Plates were incubated for 30 min on a rocking platform at 4°C. Subsequently, cells were scraped and centrifuged at 12,000 g for 10 min at 4°C. The supernatant was transferred to a new tube and the protein concentration was determined using Bradford assay. Protein (1.5 mg) was loaded on a sucrose gradient. A continuous gradient was prepared from 10% to 40% sucrose using an ultracentrifuge tube with a total volume of 4 mL. Lysates were distributed in the sucrose gradient using ultracentrifugation (194,000 g, at least 16 h, Beckman SW55 Ti rotor, Brea, California, USA). After centrifugation, each sample was divided into 12 fractions and taken up in 5x loading buffer. Samples were analyzed by immunoblot. Quantification was performed by determination of the relative intensities of PLK1 positive signals. The percentage of PLK1 in a certain fraction was calculated by determining the ratio between the relative intensity in a single lane and the relative intensity of the sum of all PLK1-positive signals. The percentage of PLK1 in either the lysosomal or the nuclear fraction was calculated by addition of normalized PLK1 in LAMP2 or H3F3 and LMNA positive lanes, respectively.

### Immunofluorescence microscopy

For all immunofluorescence experiments, cells were washed in PBS and fixed with 4% paraformaldehyde in PBS for 20 min at room temperature. After washing the cells 3 times with PBS, permeabilization was performed as indicated. Cells were washed in PBS and blocked with 0.3% bovine serum albumin (Carl Roth, 8076.5) or 0.3% FCS in PBS, as indicated. Hoechst 33342 (end concentration 1 µg/mL; Invitrogen, H3570) was added and incubated for 30 min in the dark at room temperature. Cells were mounted with Mowiol 4–88 (Carl Roth, 07131) solution, which was prepared according to the manufacturer's instructions including DABCO (1,4-diazabicyclo[2.2.2]octane; Sigma-Aldrich, D27802) supplemented with 10% n-propyl-gallate (NPG; VWR International, 8.205.990.100), and analyzed using fluorescence microscopy.

For colocalization analysis of MTOR-LAMP2, MTOR-PLK1, and RRAGC-LAMP2 permeabilization was performed with 0.1% Triton X-100 (Sigma Aldrich, 93443) in PBS for 1 min and cells were blocked with 0.3% FCS in PBS. Z stack images were taken with an AxioObserver Z1 compound microscope (Carl Zeiss, Oberkochen, Germany) with an Apotome, 63x objective (Carl Zeiss, Oberkochen, Germany) and an AxioCam MRm3 CCD camera (Zeiss, Oberkochen, Germany). For quantitative analysis, 4 or 5 representative fields of view were captured for each condition with identical exposure times and the same magnification. The Pearson correlation coefficient was calculated across raw files, without any image processing, using the colocalization module of the Zen software (Zen2012 blue edition software, Zeiss) after automatically setting the threshold with the Costes method. For presentation in figures, single layers of representative raw Z stacks were exported as TIFF with no compression using Zen2012 blue edition software (Zeiss) and brightness or contrast were adjusted, for better visibility. Brightness or contrast adjustment was not performed before quantification, and thus did not influence image quantification.

PLK1 staining in mitotic cells was performed after prometaphase arrest and a release of 30 min in full medium. Mitotic cells were collected by centrifugation (500 g, 4 min). PFA-fixed cells were permeabilized with 0.1% Triton X-100 in PBS for 1 min and cells were blocked with 0.3% FCS in PBS.

To monitor autophagosomes and autolysosomes an mRFP-GPF-LC3 tandem construct^61^ (Addgene, plasmid 21074, gift from Tamotsu Yoshimori). Cells were grown on coverslips, and transfected with the mRFP-GFP-LC3 plasmid. After 48 h cells were fixed as described above. Permeabilization was performed with 0.1% Triton X-100 in PBS for 30 to 45 s and 0.3% bovine serum albumin was used for blocking. Images were taken with an AxioImager Z1 compound microscope from Zeiss, 63x objective and an AxioCam MRm3 CCD camera. Prior to quantification images were deconvoluted using Huygens software, Huygens remote manager v3.0.3 (Scientific Volume Imaging). For image parameters a pixel size of 60 nm was assumed. For processing parameters the classic maximum likelihood estimation deconvolution algorithm was chosen and the signal/noise ratio was set to 90 for all channels. The number of green and red puncta was counted using the spot detection function of Imaris Version 7.7.2 (BITPLANE AG). The background subtraction was ticked. As filter type, quality above threshold was chosen. Within one experiment the threshold and the estimated xy diameter were kept equal for all analyzed images. To determine the percentage of autolysosomes per cell, we counted red and green puncta, and subsequently calculated the difference between mRFP and GFP puncta, which we expressed as the percentage of all red puncta per cell. At least 25 nonmitotic cells, as judged by Hoechst staining, were counted per condition. For presentation in figures, representative raw images were exported as TIFF with no compression using Zen2012 blue edition software (Zeiss) and brightness or contrast were adjusted, for better visibility. Images are shown without prior deconvolution. Brightness or contrast adjustment was not performed before quantification, and thus did not alter the numbers of quantified green and red puncta.

For the SQSTM1 staining, permeabilization was performed with 1% saponin (Sigma Aldrich, 47036) for 15 min and cells were blocked with 0.3% FCS in PBS. Z stack images were taken with an AxioObserver Z1 compound microscope with Apotome from Zeiss, 63x objective, AxioCam MRm3 CCD camera. For quantitative analysis, at least 5 representative fields of view were captured for each condition with identical exposure times and the same magnification. The total area of SQSTM1/p62 positive foci was calculated using ImageJ 1.47v. The threshold was set manually and kept identical for comparative analysis before applying the “Analyze Particles” function. The total area was normalized to the number of nuclei.

For presentation in figures, maximum intensity projections of representative raw images were exported as TIFF with no compression using Zen2012 blue edition software (Zeiss) and brightness or contrast were adjusted, for better visibility. Brightness or contrast adjustment was not performed before quantification, and thus did not influence image quantification.

### *C. elegans* experiments

The *C. elegans* strain MAH14 (*daf-2(e1370); adIs2122[lgg-1p::gfp::lgg-1 +rol-6]**^64^*) was used for this study. The strain was maintained at 20°C and raised on NGM plates seeded with *Escherichia coli* strain OP50 as described previously.^75^ To investigate autophagy, eggs from MAH14 animals were transferred to RNAi plates (*atg-18* RNAi clone was from the Vidal library,^76^ and *plk-1* RNAi clone was from the Ahringer library^77^) and incubated at 25°C to induce dauers. Following incubation for 6 d, dauers were anesthetized with sodium azide, arranged vertically on agar plates and imaged using an AxioImager Z1 compound microscope fitted with an AxioCam MRm3 CCD camera. GFP intensity was quantified using Image J software and normalized to the size of the animals.

### Statistics

Quantifications of experiments were displayed and statistically analyzed using GraphPad Prism Version 5.00. For all experiments in human cells, the mean and the standard error of the mean (SEM) were plotted. For quantification of GFP::LGG-1 fluorescence in *C. elegans* the mean and the standard deviation (s. d.) were plotted. Two groups were compared using a nonparametric 2-tailed Student *t* test assuming unequal variances. For comparison of multiple groups, a one-way ANOVA followed by the Bonferroni multiple comparison test was used. *P* values below 0.05 were considered significant.

## Supplementary Material

Supplementary files

## Abbreviations

aa: amino acids
AMPK: AMP-activated protein kinase
ATG: autophagy-related
ATP: adenosine triphosphate
BafA: bafilomycin A_1_
BSA: bovine serum albumin
CCNE1: cyclin E1
*C. elegans*: *Caenorhabditis elegans*
CID: collision-induced dissociation
d: days
DABCO: 1,4-diazabicyclo[2.2.2]octane
DMEM: Dulbecco's modified Eagle's medium
DNA: deoxyribonucleic acid
GAP: GTPase activating protein
GAPDH: glyceraldehyde-3-phosphate dehydrogenase
GFP: green fluorescent protein
H3F3: H3 histone family member 3
HBSS: Hank's balanced salt solution
IB: immunoblot
IF: immunofluorescence
INSR: insulin receptor
IP: immunoprecipitation
KA: kinase assay
LAMP2: lysosomal associated membrane protein 2
LMNA: lamin A/C
MAP1LC3A/LC3A: microtubule associated protein 1 light chain 3 α
mRFP: monomeric red fluorescent protein
MS: mass spectrometry
MTOR: mechanistic target of rapamycin
MTORC1: MTOR complex 1
MTORC2: MTOR complex 2
ns: nonsignificant
PBS: phosphate-buffered saline
PE: phosphatidylethanolamine
PLK1: polo like kinase 1
RHEB: Ras homolog enriched in brain
RICTOR: RPTOR independent companion of MTOR complex 2
RNA: ribonucleic acid
RNAi: RNA interference
RPS6: ribosomal protein S6
RPS6KA1/RSK: ribosomal protein S6 kinase A1
RPS6KB (p70)/p70-S6K: ribosomal protein S6 kinase B 70 kDa
RPS6KB (p85)/p85-S6K: ribosomal protein S6 kinase B 85 kDa
RPTOR/RAPTOR: regulatory associated protein of MTOR complex 1
RRAGC/RAGC: Ras related GTP binding C
s. d.: standard deviation
SDS: sodium dodecyl sulfate
SEM: standard error of the mean
shRNA: small hairpin RNA
SILAC: stable isotope labeling by amino acids in cell culture
siRNA: small interference RNA
SQSTM1/p62: sequestosome 1
TSC1: tuberous sclerosis 1
TSC2: tuberous sclerosis 2
ULK1: unc-51 like autophagy activating kinase 1
vs: versus
