# Supplementary material for: PLK1 (polo like kinase 1) inhibits MTOR complex 1 and promotes autophagy
Source: Autophagy. 2017 Jan 19;13(3):486–505. doi: 10.1080/15548627.2016.1263781 (PMC5361591; doi:10.1080/15548627.2016.1263781)
Supplement: Supplementary files [file kaup-13-03-1263781-s001.zip › 2015AUTO0489R5-s03.docx]

**Supplemental figure legends for** **Ruf et al.:**

**PLK1 (polo like kinase 1) inhibits MTOR complex 1 and promotes autophagy**

**Figure S1.** PLK1 interacts with MTOR. (**A, B, C**) MS data were extracted from Table S4 and dataset PXD001244 of the ProteomeXchange database (http://proteomecentral.proteomexchange.org) of Schwarz et al.^43^, for detailed analysis of PLK1-specific SILAC peptides in MTOR immunoprecipitations (IP) versus control (mock) IP. (**A**) PLK1 identification by MS-based analysis of MTOR interactors in HeLa cells.^43^ Shown is the full-length amino acid sequence of PLK1 with six identified peptides in red. Two peptides are overlapping (FSIAPSSLDPSNR and KPLTVLNK). Sequence coverage: 11%. (**B**) Zoomed MS1 spectrum showing the protonated peptide IGDFGLATK of PLK1 in a SILAC experiment. Observed is the “heavy”-labeled (MTOR IP) peptide, while no distinctive isotopic pattern is present for the corresponding “light”-labeled (mock IP) peptides. Thus, PLK1 was found to be specifically enriched with MTOR. The star indicates the peptide peak which was subjected to collision-induced dissociation (CID) for identification. (**C**) Annotated MS2 spectrum of the peptide IGDFGLATK of PLK1. (**D**) HeLa cells were cultured in full medium. Immunoprecipitation (IP) was performed with PLK1 and control (mock) antibodies. Samples were analyzed by immunoblotting. Data shown are representative of n=3 independent experiments. (**E**) *RPTOR* (sh*RPTOR*) or control (shControl) sh RNA HeLa cells were cultured in full medium. Immunoprecipitation (IP) was performed with PLK1 and control (mock) antibodies. Samples were analyzed by immunoblotting. Data shown are representative of n=4 independent experiments. (**F**) Quantification of IP samples shown in (**E**). Ratio of MTOR:PLK1 was calculated for n=4 independent experiments. Data are normalized to 1 for shControl condition and represented as mean ± SEM. A nonparametric two-tailed Student t test was applied; ns, nonsignificant.

**Figure S2.** PLK1 regulates autophagy in interphase cells. (**A**) HeLa cells were starved for 1 h for amino acids (aa) and growth factors, treated with BI2536 for 30 min prior to lysis, and stimulated with amino acids and insulin (ins) for 35 min. Mitotic cells were collected from sh*PLK1* knockdown cells. Samples were analyzed by immunoblotting. Data shown are representative of n=3 independent experiments. aa, amino acids; ins, insulin. (**B**) HeLa cells were treated with BI2536 for 16 h. Samples were analyzed by immunoblotting. Data shown are representative of n=3 independent experiments. (**C**) *PLK1* (sh*PLK1*) or control (shControl) shRNA cells were induced for 48 h with doxycyline and mitotic cells removed by shake-off. Samples were analyzed by immunoblotting. Data shown are representative of n=3 independent experiments. (**D**) HeLa cells were starved for 1 h for amino acids and growth factors, stimulated with amino acids and insulin for 35 min, followed by 30 min amino acid starvation. Media were supplemented with bafilomycin A_1_ (BafA) as indicated. Data shown are representative of n=3 independent experiments. (**E**) HeLa cells were arrested in mitosis by consecutive aphidicolin and nocodazole treatment and released for the indicated times, up to 4 h. Samples were analyzed by immunoblotting. Data shown are representative of n=3 independent experiments. (**F**) Schematic overview of the autophagy assay in *C. elegans*. Eggs from *daf-2; GFP::LGG-1* mutants were transferred to RNAi plates and incubated at 25°C to induce *dauers* for 6 days before microscopy analysis of GFP intensity. (**G**) HeLa cells were treated and kinase reaction was performed as described in Fig. 1K. Samples were analyzed by immunoblotting. Data shown are representative of n=2 independent experiments; IP, immunoprecipitation. (**H**) Model of the 2 separate mechanisms of PLK1-MTORC1 (MTOR-RPTOR) crosstalk in autophagy: 1) Active PLK1 translocates MTORC1 away from the lysosome to inhibit MTORC1 and activate autophagy. 2) Amino acid starvation increases PLK1-MTORC1 interaction in the cytoplasm, and this is independent of MTOR or PLK1 kinase activity. Increased cytoplasmic PLK1-MTORC1 binding may indirectly contribute to PLK1’s inhibitory effect on MTORC1. The molecules which enhance MTORC1-PLK1 interaction in response to starvation are unknown.
